# The kinetics and transmission-blocking dynamics of *P. falciparum* sexual-stage antibody responses in a six-year cohort of Ugandan children and adults

**DOI:** 10.21203/rs.3.rs-8733488/v1

**Published:** 2026-02-09

**Authors:** Sara Lynn Blanken, Patience Nayebare, Jonathan Briese, Gerine Nijman, Karina Teelen, Rianne Stoter, Geert-Jan van Gemert, Wouter Graumans, Tate Oulton, Catriona Patterson, Abdoulie Drammeh, Marloes de Bruijni, Judith Bolscher, Lauren M. Cohee, Sanjai Kumar, Noah Sather, Alvaro Molina-Cruz, Emmanuel Arinaitwe, Moses R Kamya, Koen Dechering, Grant Dorsey, Chris Drakeley, Matthijs M. Jore, Teun Bousema, William Stone

**Affiliations:** 1: Department of Medical Microbiology, Radboud University Medical Centre, Nijmegen, The Netherlands; 2: Infectious Diseases Research Collaboration, 2C Nakasero Hill Road, Kampala, Uganda.; 3: Faculty of Infectious and Tropical Diseases, London School of Hygiene and Tropical Medicine, Keppel Street, Bloomsbury, London, UK.; 4: Medical Research Council Unit, Fajara, The Gambia; 5: TropIQ Health Sciences, Nijmegen, The Netherlands; 6: Department of Clinical Sciences, Liverpool School of Tropical Medicine, Liverpool, UK; 7: Laboratory of Emerging Pathogens, Center for Biologics Evaluation and Research, Food and Drug Administrations, Silver Spring, Maryland, USA; 8: Center for Global Infectious Disease Research, Seattle Children’s Research Institute, Seattle, WA, USA; 9: Laboratory of Malaria and Vector Research, National Institute of Allergy and Infectious Diseases, National Institutes of Health, Rockville, MD, USA; 10: Department of Medicine, Makerere University College of Health Sciences, Kampala; 11: Department of Medicine, San Francisco General Hospital, University of California, San Francisco, USA

## Abstract

Individuals naturally infected with *Plasmodium* can develop antibodies against the parasite’s sexual stages that may inhibit transmission when ingested during a mosquito’s blood meal. This study aims to unravel the immune signature and longitudinal dynamics of naturally occurring transmission reducing activity (TRA). Using data from 611 standard membrane feeding assays and up to 18 assessments per individual, we show that TRA occurs naturally and may persist over time in a minority of naturally exposed individuals. We also show that history of asymptomatic parasite exposure is positively related to TRA and to antibodies against *P. falciparum* sexual-stage antigens. Antibodies against Pfs48/45 and Pfs230 appeared relatively short-lived and predicted high-level TRA. Finally, we find that naturally acquired antibodies against thirteen other sexual-stage antigens (including Pfs47, Pf77, PfHAP2 and PfMDV1) were associated with high-level TRA. Together, our analysis shows that naturally acquired sexual-stage immune responses are dynamic and can inhibit onward parasite transmission.

## Introduction

An estimated 263 million people suffered from malaria in 2023, resulting in approximately 597,000 deaths [1]. Naturally acquired protective immunity develops following repeated exposure, attenuates multiplication and sequestration of pathogenic asexual parasites, and is associated with improved clinical outcome and asymptomatic parasite carriage. Natural exposure to malaria parasites may also result in immune responses to parasite transmission stages; this antibody-mediated immunity can affect the parasite’s transmission to mosquitoes.

Malaria spreads from humans to mosquitoes through sexual stage *Plasmodium* gametocytes. Gametocytes originate from a minority of asexual parasites that undergo sexual differentiation, maturing into dimorphic female or male gametocytes. When an *Anopheles* mosquito ingests gametocyte-infected blood, female and male gametocytes egress from red blood cells (RBCs) as macro- (female) and micro- (male) gametes. Upon fertilization, zygotes are formed that develop into oocysts on the mosquito’s midgut. Mature oocysts release sporozoites that migrate to the salivary glands, enabling transmission during subsequent blood-meals. Higher gametocyte densities increase mosquito infection likelihood, yet transmission efficiency varies widely with some high-density gametocyte carriers failing to infect mosquitoes in feeding assays [2, 3]. Replacing plasma from infected blood with malaria-naïve serum increases mosquito infection rates [4, 5], while serum or purified immunoglobulins from malaria-exposed individuals can also reduce *P. falciparum* transmission in standard membrane feeding assays (SMFA) [6, 7]. These observations suggest a role for humoral immunity in mediating natural transmission of *Plasmodium* parasites.

The best characterized sexual-stage antigens are Pfs48/45 and Pfs230, that are promising transmission-blocking vaccine candidates [8–12]. Expressed on mature gametocyte surfaces, these proteins remain intracellular (within RBCs) while circulating in the blood. Following gamete egress in the mosquito midgut, the antigens become exposed, and Pfs230 forms a surface complex with Pfs48/45 [13–15]. Pfs48/45 and Pfs230 are essential for establishing mosquito infection [16] [17]. Naturally acquired antibodies against Pfs48/45 and Pfs230 develop and have no access to ‘live’ antigens while gametocytes circulate. However, antibodies co-ingested during mosquito feeding can bind the exposed antigens and inhibit infection. The density and functionality of naturally occurring antibodies against Pfs48/45 and Pfs230 are likely influenced by gametocyte exposure duration and intensity [18, 19] but their acquisition following cumulative exposure [19–22] does not necessarily result in increasing transmission-reducing activity (TRA). Pfs48/45 and Pfs230 responses have been associated with functional TRA in feeds supplemented with purified antibodies against specific antigenic regions or domains [20], and in indirect analyses that correlated serum antibody levels with TRA of this serum sample or purified total immunoglobulins [19, 20, 23–29]. Importantly, positive correlations between Pfs230/Pfs48/45 antibody densities and serum TRA were not observed in all indirect analyses [30, 31] and TRA does not appear to increase with age [27, 32]. Despite robust linkage to naturally occurring TRA, capturing the role of sexual-stage antibodies in malaria transmission at the population level remains challenging.

Prior work has identified individuals with transmission-reducing immunity who do not have detectable antibodies against Pfs48/45 and Pfs230 [19, 20, 29, 30], suggesting that antibodies against additional antigens may contribute to transmission reduction. Several less well-characterised antigens have been proposed as potential transmission blocking vaccine candidates [33–36]. These include Pfs47, a paralog of Pfs48/45 expressed on female gametes, zygotes, and ookinetes [36]; PfHAP2, a hapless 2 family fusogen essential for fertilization [37]; mdv1 (male development gene 1), an asexual-stage protein essential for the production of male gametocytes during sexual differentiation [33, 38]; and Pf77, a sexual-stage antigen with an unknown function and pan developmental expression [33]. Little is known about the role of Pfs47, Pf77, PfHAP2and PfMDV1 in naturally occurring TRA.

This study aims to provide a comprehensive understanding of the humoral correlates of potent *P. falciparum* transmission reduction. Using plasma samples from Ugandans participating in a 6-year longitudinal study with carefully characterized malaria exposure, we assessed the characteristics and patterns of naturally occurring immune responses against sexual and asexual-stage antigens in relation to parasite exposure. We also studied antibody responses against Pfs48/45, Pfs230, Pfs47, PfHap2, PfMDV1 and Pf77 in relation to functional TRA. In addition, we examined the relationship between TRA and antibody responses against a panel of gametocyte-expressed proteins detected by microarray, aiming to uncover novel correlates of effective transmission reducing immunity. Finally, we explored the genesis, maintenance, and serological correlates of naturally acquired TRA using longitudinal SMFA experiments spanning multiple years and malaria episodes.

## Results

Between October 2011 and September 2017, an observational cohort study was conducted in Nagongera subcounty in Tororo District, Eastern Uganda. 100 households were enrolled and all children 0.5–10 years at the time of recruitment were invited to participate in the study, along with 1 primary caregiver (>18 years) [39]. At enrolment, 19.8% (n/N=97/491) of participants carried microscopically detected *P. falciparum* parasites and 5.5% (n/N=27/491) had uncomplicated malaria. Participants were actively followed every three months (October 2011-November 2014) or monthly (December 2014-September 2017). During each visit, blood samples were collected to assess parasite presence microscopically and molecularly using loop mediated isothermal amplification (LAMP). Participants also had access to care 7 days/week in case symptoms developed (passive follow-up). Malaria was defined as a parasite-positive thick blood smear with documented fever (tympanic temperature > 38.0 °C) or history of fever in past 24 hours. Malaria cases were managed according to national guidelines [40]; asymptomatic parasite carriage was not treated.

### Antibody responses against different sexual-stage antigens are naturally acquired and intercorrelate

**Figure 1.**
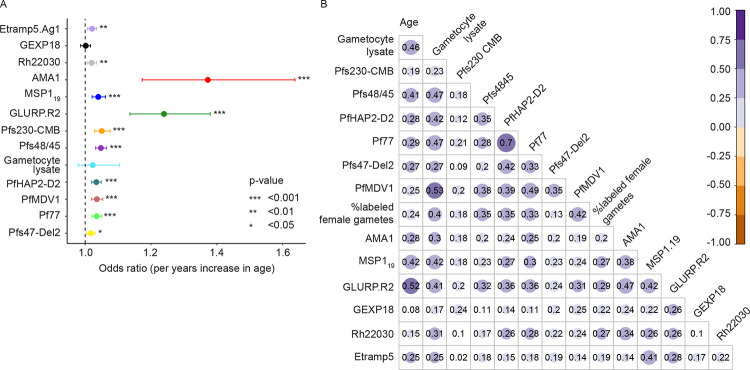
Antibodies against sexual-stage antigens are associated with each other and with participant age. a) Forest plot depicting odds ratios (OR) and corresponding 95% confidence intervals (CI) of seroprevalence changing per years increase in participant age. Sexual-stage antibody responses (against Pfs230-CMB, Pfs48/45, Gametocyte lysate, PfHAP2-D2, PfMDV1, Pf77, Ps47-Del2) were quantified using ELISA and samples were seropositive if +2SD above the mean. Asexual-stage antibody responses (against Etramp5.Ag1, GEXP18, Rh22030, AMA1, MSP1.19, GLURP.R2) were quantified using Luminex and samples were considered seropositive if their intensity was +3SD above the mean. Odds ratios were calculated per antibody response using general linear models with age (in years) as predictor and seropositivity as outcome variable, assuming a binomial distribution. P-values are indicated by asterisks. b) Matrix showing spearman correlations between antibody responses and participant age (in years). Colours and text indicate spearman rho’s; a white cell indicates that the association was not statistically significant (p > 0.05). ab) Samples from the 2013–2014 cross section were selected (n=313 samples, N=313 individuals).

We first assessed whether antibody prevalence to sexual and asexual parasite antigens was associated with participant age. For this, a cross-section of samples was selected during a period of high transmission intensity (2013–2014), with a single sample selected per cohort participant (N=313). Antibodies against asexual-stage antigens associated with cumulative (AMA-1, MSP1_19_, GLURP.R2) or recent (Etramp5.Ag1, GEXP18, Rh22030) parasite exposure were quantified using a Luminex bead-based assay. Antibody responses were generally highest for AMA-1, with a seroprevalence of 70.4% (n/N=50/71) in children aged below 5 years and 98.8% (n/N=85/86) in individuals aged 18 years or older (adults). The odds of being seropositive increased with age for antibodies associated with cumulative exposure (all P<0.001, GLM) and for antibodies against Etramp5.Ag1 (P=0.0022, GLM) and Rh22030 (P=0.0076, GLM, Figure 1A) but not GEXP18 (P=0.832, GLM).

Antibody levels against recombinant sexual-stage antigens and against *P. falciparum* gametocyte extract were measured by ELISA. The odds of being seropositive increased with participant age for all recombinant sexual-stage antigens (Figure 1A, all P<0.014, GLM) but not gametocyte extract (P=0.426, GLM). Amongst purified sexual-stage antigens, responses were lowest for PfHAP2-D2 and highest for Pfs230-CMB (Supplementary Table 1). Antibodies against both sexual-stage and asexual-stage antibody levels were generally positively correlated with each other and with antibody binding to female gametes measured by surface immune-fluorescence assay (SIFA) (all ρ≥0.18, all P<0.025, Spearman, Figure 1B).

### Sexual-stage antibody responses generally decrease as transmission intensity declines

**Figure 2.**
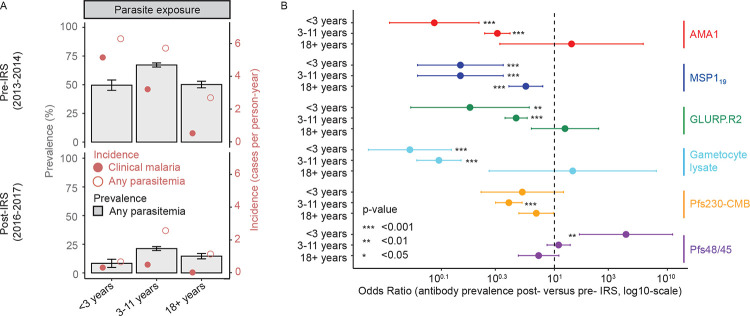
Sexual-stage antibody responses are associated with declines in transmission intensity. a) Parasite prevalence (left y-axis) and incidence of infections (right-axis) per age category before (top) and after (bottom) the implementation of indoor residual spraying (IRS) in December 2014. Parasite prevalence is calculated as the number of parasite positive (by blood smear or LAMP test) visits divided by the total number of visits. Incidence rates were calculated by identifying unique infections per individual, defined by parasite positive visits (Any parasitaemia, open dots) or clinical malaria cases specifically (Clinical malaria, filled dot). An infection was considered unique when it was separated from another infection by more than 14 days. Incidence was estimated as the number of infections or malaria episodes divided by total person-time (in person-years), stratified by period (pre- and post-IRS) and age category. b) Forest plot showing odds ratios (ORs) for antibody seropositivity after versus before implementation of IRS, stratified by age category. Per age category, odds ratios were calculated using generalized estimating equations (GEE) with a logit link and binomial distribution for every antibody response separately. Samples from 2013–2014 (pre-IRS, n=1113) and 2016–2017 (post-IRS, n=847) were selected (n=1960, N = 433 individuals). ab) Age categories were defined so that individuals in the youngest group (<3 years of age) in the post-IRS survey were born under the protection of IRS (n=181 samples, N=83 individuals).

We next compared parasite exposure and sexual-stage antibody responses by age, before and after a decline in transmission following effective implementation of indoor residual spraying (IRS) that reduced the entomologic inoculation rate (EIR) from 310 infected bites per person per year before IRS to 12.4 in the first year following IRS [41]. We analysed samples taken in 2013–2014 (pre-IRS) and in 2016–2017 (post-IRS), classifying children under 3 years of age in the post-IRS survey as having been born under the protection of IRS. Parasite prevalence and the incidence of infections (clinical and non-clinical) were markedly lower in the period post-IRS compared to that pre-IRS (Figure 2A).

Asexual antibody prevalence appeared stable in adults despite the decrease in parasite exposure, with significant reductions in seropositivity post-IRS for MSP1_19_ only (OR = 0.56, 95% CI = 0.40–0.79, P_FDR_ <0.001, GEE, Figure 2B). The odds of being seropositive as an adult post-IRS decreased for sexual-stage antibodies against Pfs230-CMB and Pfs48/45 (Pfs230-CMB: OR = 0.70, 95% CI = 0.48–0.99, P_FDR_=0.061, Pfs48/45: OR = 0.73, 95% CI = 0.48–1.10, P_FDR_=0.130, GEE), though both effects were not statistically significant. For the 3–11-year-old group, seropositivity for asexual-stage antigens (AMA-1, MSP1_19_, and GLURP.R2) was significantly lower (all P_FDR_ <0.001, GEE). A similar effect was seen in the 3–11-year-old group for antibodies against Pfs230-CMB and gametocyte lysate (Pfs230-CMB: OR = 0.40, 95% CI = 0.30–0.52, P_FDR_<0.001, gametocyte lysate: OR = 0.10, 95% CI = 0.06–0.15, P_FDR_<0.001).

As expected, children <3 years old in the post-IRS survey had markedly lower odds of being seropositive for antibodies against AMA-1, MSP1_19_ and GLURP.R2 compared to children of the same age-group prior to IRS implementation (all OR < 0.18, all P_FDR_<0.001, GEE, Figure 2B). A similar effect was observed for antibodies against gametocyte lysate (OR = 0.05, 95% CI = 0.02–0.12, P_FDR_<0.001, GEE). For antibodies against Pfs230-CMB the odds of seropositivity were also lower post-IRS, though the effect was not statistically significant (OR = 0.52, 95% CI = 0.22–1.21, P_FDR_=0.128, GEE). In this age group, the only antigen for which the odds of being seropositive were significantly greater in post-IRS samples was Pfs48/45 (OR = 4.30, 95% CI = 1.66–11.1, P_FDR_=0.008, GEE) and driven by a very low antibody prevalence pre-IRS in this age group (7.3% (9/123) pre-IRS; 25.8% (16/62) post-IRS).

### Sexual-stage antibody prevalence is associated with intensity of prior exposure and varies with symptomatic status

**Figure 3.**
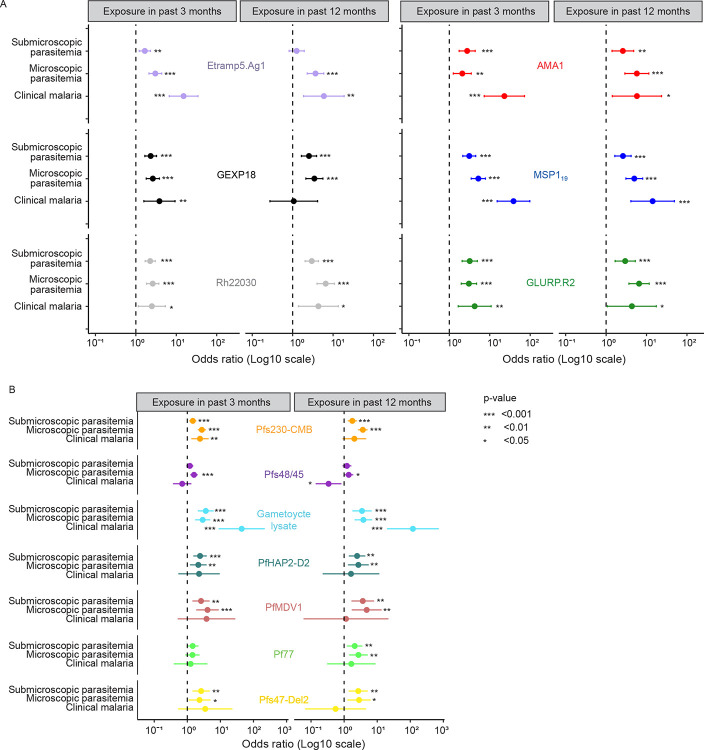
Seroprevalence of antibodies against sexual-stage antibodies is related to history of parasite exposure. a,b) forest plots showing associations between previous parasite exposure and asexual (a) and sexual-stage (b) antibody seropositivity. Odds ratios were calculated using generalized linear models, with seropositivity status for each antibody response at timepoint X as the outcome variable and history of exposure as predictor variables, separated by infection type. For asymptomatic infections, parasite exposure was calculated per participant as the number of visits with sub microscopic parasitaemia (microscopy negative but LAMP positive) or microscopic parasitaemia, expressed as proportion of the total number of scheduled visits for that participant and time-frame (i.e. a proportion ranging from 0–1 in the 3 or 12 months before timepoint X). Clinical incidence was calculated as the sum of new clinical episodes (separated by at least 14 days) divided by the person-months of observation for a given participant and time-frame. Infection categories are depicted on the y-axis; x-axis shows odds-ratios (Log10-scale) per 50% increase in parasite prevalence for asymptomatic infections and per one additional clinical malaria episode per person-month for clinical malaria. n=2600 samples with antibody observations (from 2013–2017), N = 433 participants, p-values are indicated with asterisks.

The association between individual-level parasite exposure and antibody response was explored in greater depth by assessing individual-level exposure to clinical, asymptomatic microscopically detectable, or asymptomatic sub-microscopic (microscopy negative but LAMP positive) infections, assessed over three-month and 12-month time-frames. For a 50% increase in the proportion of visits with asymptomatic parasitaemia (microscopically detectable or sub-microscopic) in the past three or 12 months, the odds of seropositivity for asexual parasite antibodies significantly increased for recent exposure markers Etramp5.Ag1 and Rh22030, and for cumulative exposure markers AMA1 and MSP1_19_ and GLURP.R2 (Figure 3A).

For sexual-stage responses, an increased proportion of visits with asymptomatic microscopically detectable parasites in the past three or 12 months was associated with significantly higher odds of seropositivity for Pfs230-CMB, Pfs48/45, and gametocyte lysate (Figure 3B). Increased asymptomatic sub-microscopic infections were also associated with increases in seropositivity for Pfs230-CMB and gametocyte lysate, while showing no association with Pfs48/45 seropositivity. Similarly, increased incidence of clinical infections was associated with higher seropositivity for Pfs230-CMB (three months only) and gametocyte lysate (three and 12 months). However, in contrast to all other asexual and sexual-stage antigens, increased incidence of clinical malaria in the past 12 months was significantly associated with a 66% decrease in the odds of being seropositive for antibodies against Pfs48/45 (OR=0.34, P_FDR_=0.027, GLM). A higher frequency of visits with sub-microscopic or microscopically detectable parasites in the past three or 12 months was also positively associated with seropositivity for PfHAP2-D2, PfMDV1, and Pfs47-Del2 (Figure 3B). For Pf77, this effect was only significant when a 12-month period of exposure was considered.

### Sexual-stage antibodies show decay rates more akin to markers of recent exposure than historic exposure

**Figure 4.**
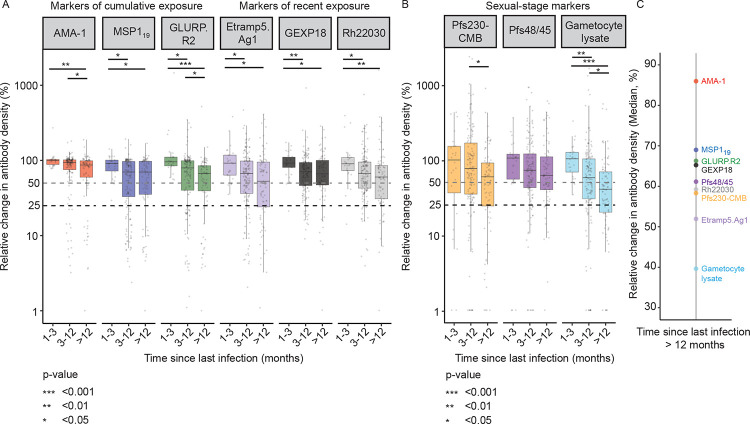
Relative sexual-stage antibody densities relate to time since last parasite exposure. a,b) Box plots showing relative asexual (a) and sexual (b) stage antibody densities (mean and interquartile ranges). Relative antibody densities were calculated per antibody response as the percent change in antibody density between each parasite-free time-point and its most recent parasite-positive time-point (i.e. antibody decay). The last parasite-positive time-point included visits with asymptomatic parasitaemia (either sub-microscopic or microscopic) as well as clinical malaria episodes. The time since last parasite-positive time-point was categorized into groups of samples with recent (1–3 months since positivity), intermediate (3–12 months) or historic exposure (>12 months), indicated on the x-axis. The y-axis depicts the relative change in antibody density (in percentages), a 50% and 25% relative reduction are indicated by a grey and black dashed line, respectively. c) A dot plot showing median relative antibody densities (in percentages) per antibody response (asexual and sexual-stage responses) for samples with historical exposure only (> 12 months since the last parasite positive visit). n=2600 samples with antibody observations (from 2013–2017), N = 433 participants, p-values are indicated by asterisks.

To explore longevity of sexual-stage antibody responses, we next assessed the relationship between antibody density and time since last infection, categorising samples into groups with recent (1–3 months since infection), intermediate (3–12 months) or historic exposure (>12 months). Antibody decay in the absence of re-exposure was expressed as the percent change in antibody density between each parasite-free time-point and its most recent parasite-positive time-point (i.e. relative antibody density). All asexual-stage antibodies showed significant relative decreases in antibody density with time since infection (Figure 4A, Supplementary Table 2). These decreases were greater and more rapid for Etramp5.Ag1 than for classic markers of cumulative exposure; for samples with historic but no (confirmed) recent infection exposure, relative antibody density was 86.0% for AMA1 (IQR 58.8–98.1) compared to 51.9% for Etramp5.Ag1 (IQR 23.1–94.0, P<0.001, Wilcoxon signed-rank test). For sexual-stage antibody responses, considerable decreases were observed for both Pfs48/45 and Pfs230-CMB when the last parasite exposure was >12 months ago (relative density 58.3%, IQR 23.3–89.7, P_FDR_=0.108 for Pfs230-CMB, and 61.2%, IQR 39.1–108.6, P_FDR_=0.137 for Pfs48/45, Mann-Whitney U test) (Figure 4B). A decrease in antibody density was also observed for antibodies against gametocyte extract when exposure was >12 months ago (median relative density 39.6%, IQR 19.1–68.2). When antigens were ranked based on median reductions in antibody density following 12 months of no parasite exposure, antibody reductions were modest for markers of cumulative exposure whilst sexual-stage antigens showed large reductions that were similar to those observed for markers of recent exposure (Figure 4C).

### Sexual-stage antibody responses are predictive of the naturally occurring transmission reducing activity

**Figure 5.**
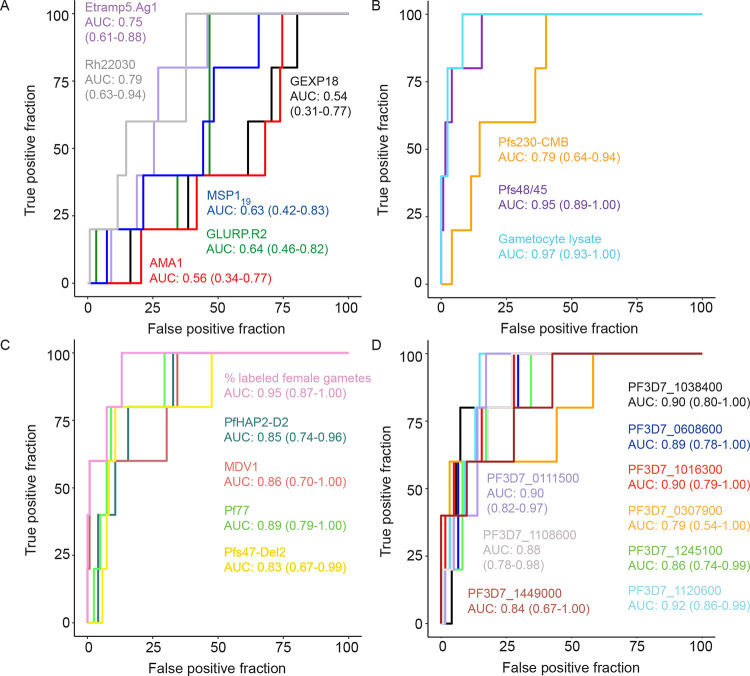
Antibody levels against sexual-stage *P. falciparum* antigens predict malaria transmission blockade. Receiver operating characteristic (ROC) plots showing the performance of asexual (a,b) and sexual-stage (c,d) antibody densities in classifying functional transmission reducing activity. Transmission reducing activity (TRA) was determined by standard membrane feeding assays that compare oocyst densities in mosquitoes fed on *P. falciparum* gametocyte bloodmeals supplemented with field-sample isolated IgGs with oocyst densities from control feeds without isolated IgGs. A threshold of 80% reduction in oocyst density was set as high-level TRA (i.e. transmission blockade) and tested against samples with low-level TRA (−50% to 9% TRA). The lower limit of TRA was set to exclude a potential effect of transmission enhancing samples. Samples from a 2013–2014 cross-sectional dataset were used to avoid bias from correlated measurements within individuals and we only used samples with complete antibody data so that all ROC plots are based on the same dataset (n=122 samples with −50% to 9% TRA, n=5 samples with ≥80% TRA). Area under the curve (AUC), together with the 95% confidence intervals (CI) are indicated in text for each antibody response separately.

We next evaluated the association between specific sexual-stage antibody densities and the level of TRA when purified total IgG was mixed with mature gametocytes and offered to mosquitoes in an SMFA. For this, one sample per individual in the 2013–2014 cross-section (n=313) was assayed. Only 5 participants (1.60%; n/N=5/313) achieved ≥80% reductions in oocyst formation in mosquitoes and were defined as ‘transmission blockers’. The discriminative power of antibody levels to predict this high and robust level of TRA [6, 42, 43] was assessed with receiver operating curves (ROCs) that compared samples with high-level TRA (TRA≥80%, n=5) with samples with negligible activity (TRA of −50% to 9%, n=122, Table 1). The lower limit of TRA was set to exclude the potential influence of transmission enhancing samples [44] (n=13 samples). The weakest predictors of TRA≥80% were antibodies against GEXP18 and PfAMA1 (both AUC<0.60, Figure 5A). The strongest predictors were antibodies against gametocyte lysate (AUC 0.97, 95% CI: 0.93–1.00), followed by antibodies against Pfs48/45 (AUC 0.95, 95% CI: 0.89–1.00) and antibody binding to the surface of intact female gametes measured by SIFA (AUC 0.95, 95% CI: 0.87–1.00, Figure 5C) with no significant differences between the three (P>0.626, DeLong’s test). Antibodies against Pf77, Pfs47-Del2, PfHAP2-D2 and PfMDV1 were all similarly reasonable predictors of high-level TRA (all AUC>0.80, all P>0.591, DeLong’s test, Figure 5C). Amongst asexual responses, antibodies against Etramp5.Ag1 and Rh22030 – both markers of recent exposure - predicted high-level TRA best; their performance did not differ significantly from antibodies against Pfs230-CMB, the weakest predictor amongst sexual-stage antigens (Both P>0.664, DeLong’s test). The discriminative power of antibody levels to predict high-level TRA did not change when samples with intermediate TRA were included in the comparator non-blocking group (TRA ≥80% n=5, versus TRA −50 to 79%, n = 130, Supplementary Figure 1).

A protein microarray was constructed to assess responses to a wider panel of gametocyte proteins. The array comprised 588 proteins, selected for potential utility as biomarkers of gametocyte exposure or likelihood of association with transmission blocking immunity (Supplementary Table 3). Large proteins were divided into multiple fragments for expression using an *Escherichia coli* based *in vitro* transcription-translation (IVTT) system, resulting in a total of 1,135 fragments <1000 amino acids in length on the printed array.

Analysing the same 2013–2014 cross-section as above, we identified 9 gametocyte-expressed micro-proteins (represented by 10 array protein targets) that had statistically significantly higher antibody densities in participants with high-level TRA (≥ 80%) compared to individuals with low-level TRA (−50 to 9%, all t-test P_FDR_<0.047). Antibody responses previously associated with recent (GEXP18) and cumulative (PfAMA1, PfMSP1) parasite exposure were also quantified on the micro-array platform. These responses did not differ significantly between participants with low versus high-level TRA (all P_FDR_ > 0.204). The TRA-associated array proteins also had high AUC values (Figure 5D), with antibodies against PF3D7_1120600 being the strongest predictor (AUC 0.92, 95% CI 0.86–0.99). No statistically significant differences were observed between the predictive performance of Pfs48/45 antibodies and that of antibodies against any of the TRA-associated micro-array proteins (all P>0.070, DeLong’s test). In conclusion, using a cross-sectional dataset, we identified 15 sexual-stage antibody responses that predicted transmission blockade with good discriminatory value in the ROC analyses (AUC>0.80).

### Sexual-stage antibody responses partially explain longitudinal patterns in transmission reduction

**Figure 6.**
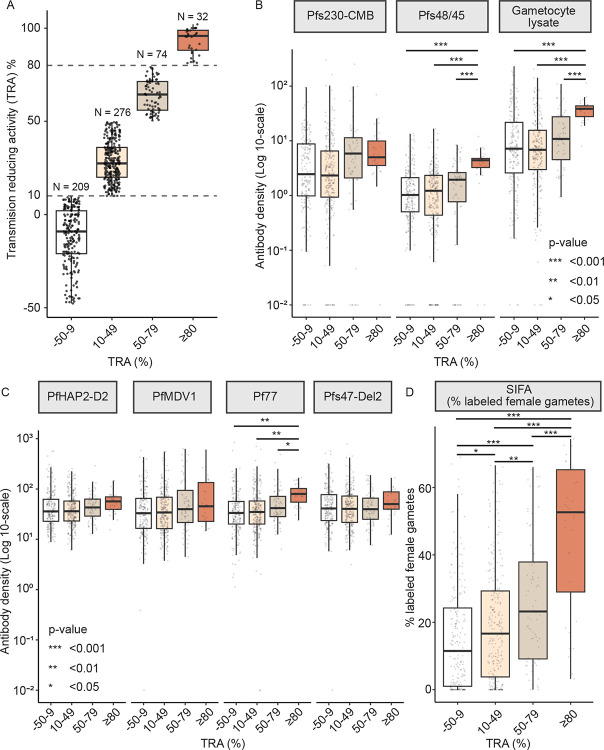
The degree of transmission reducing activity relates to sexual-stage antibody levels. a) Box plot showing the individual transmission reducing activity (TRA) measures categorized into low (−50 to 9% TRA), intermediate (10–49% and 50–79% TRA), and high-level TRA (≥80%). Sample numbers are included on top of the boxes (a total of n=591 samples, N=306 individuals); 20 samples from 18 individuals were excluded due to the potential effect of transmission enhancement (< −50% TRA). b,c) Box plots showing antibody densities (Log10-scale) against sexual-stage antigens and gametocyte lysate, stratified by TRA category. c) Box plot showing the percentage of labelled female gametes (SIFA), stratified by TRA category. P-values are indicated by asterisks.

To assess the stability of functional transmission blocking immunity and its relation with (recent) parasite exposure, we assayed additional samples from all individuals who achieved high-level TRA (≥80%) in the cross sectional survey (n=5 individuals) and ‘control’ individuals without high-level TRA in this survey (n=54 individuals, all TRA <60%) who were matched on age, household, sampling time, and infection status. For these control and blocking individuals, all available plasma samples with sufficient sample volume were processed by SMFA, ELISA and SIFA. Of the additional plasma samples from the five individuals with high-level TRA in the cross-section (n=38; 0–15 additional samples per individual), 57.9% (n/N=22/38) showed high-level TRA at other timepoints; of the additional samples from the 54 controls (n=260; 1–17 additional samples per individual), 1.92% (n/N=5/260) showed high-level TRA (≥80%) at other timepoints (p<0.001, Fisher’s exact test, Supplementary Table 4, Supplementary Figure 2), demonstrating a level of consistency of TRA estimates among samples from the same individual. A minority of ‘control’ individuals demonstrated high-level TRA (≥80%) when additional samples were tested (5 individuals, n = 70 samples of which 5 had TRA ≥80%) and will be considered transmission blockers from this point forward, alongside individuals already identified as transmission blockers at the initial cross-section (n=5, of whom 4 had multiple samples available). Whereas our threshold for high-level TRA (≥80%) was previously established for reproducibility [43], lower but functionally relevant and robust levels of TRA cannot be ruled out. We thus categorized TRA as ≥80% (32 samples), 50–79% (74 samples), 10–49% (276 samples) and −50 to 9% (209 samples, Figure 6A); excluding 20 transmission enhancing samples with a TRA of below −50%. In conclusion, this resulted in 9 individuals with repeated TRA measures that showed functional TRA (≥80%) at least once (111 samples, 3–18 samples per individual) and 46 individuals with repeated TRA measures that never showed high-level TRA (230 samples, 2–16 per individual). For all samples tested from longitudinally followed transmission-blockers (n=9 individuals, 111 samples), 27.9% (n/N=31/111) and 30.6% (n/N=34/111) fell within the highest (≥80%) and second-to-highest category of TRA (50–79%), respectively. This again suggests a level of stability of high TRA levels. For control individuals (n=46 individuals, 230 samples), only 11.6% (n/N=27/230) of samples fell within the second-to-highest category, followed by 35.7% (n/N= 81/230) and 52.8% (n/N=122/230) in the lowest two categories (−50–9% and 10–49% TRA, respectively).

Using samples tested in SMFA (n=591), median sexual-stage antibody densities were higher in high-level TRA samples compared to samples with low-level TRA (−50 to 9%) for Pfs48/45 and Pf77 (Figure 6BC, both P_FDR_<0.0022, GEE). Compared to the lower TRA categories, Pfs48/45 and Pfs230-CMB antibodies appeared higher in the intermediate TRA group (50–79%). This difference only achieved statistical significance for the recognition of activated gametes in SIFA for both the 10–49% (p=0.024) and 50–79% (p=<0.001) TRA group relative to the lowest TRA group (Figure 6D). The proportion of previous visits with asymptomatic parasitaemia was significantly higher for observations with high-level TRA (≥80%) compared to observations with low TRA (−50 to 9%, p<0.001 for both sub microscopic and microscopic infections, Table 2). In contrast, the proportion of previous visits with clinical malaria was significantly lower for observations with high compared to low TRA (p<0.001).

We next visualized the individual-level TRA trajectories in relation to history of malaria exposure for 9 individuals with high-level TRA (≥80%, Supplementary Figure 3). Six individuals had high-level TRA once; three individuals had high-level TRA at more than one occasion (i.e. blockers). For each of the three repetitive blockers, an age-matched control without evidence of high-level TRA was selected from a household in their close proximity (<1km). To support interpretation of (fluctuations in) serologic metrics across assay types, specific antibody densities and array responses (averaged for the 10 TRA associated hits) were divided into quartiles using all observations from the longitudinal blockers and controls combined (n=82, Supplementary Figure 4).

**Figure 7.**
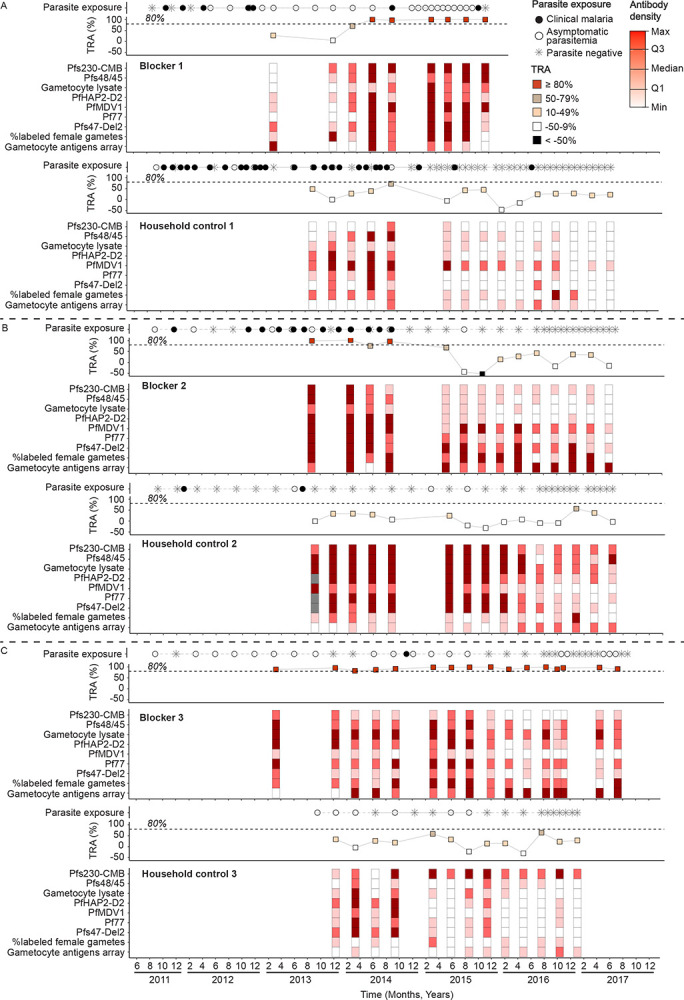
Longitudinal patterns in transmission reducing activity broadly reflect sexual-stage antibody levels. a,b,c) Transmission reducing activity (TRA) trajectories over time for six individuals: three control individuals (n=43 samples) that never reached high-level TRA (80%) and three individuals that reached the high-level TRA threshold more than once (i.e. blockers, n=39 samples). One blocker acquired high-level TRA over time (a), another lost the phenotype over time (b) and another individual continuously exceeded the high-level TRA threshold (c). Control individuals were matched to blockers based on participant age and household proximity. The maximum TRA of samples from control individuals was 71%. The lower panel shows a heatmap of antibody densities that were divided into quartiles using the 25th, 50th and 75th percentile value per antibody response. The middle panel shows TRA trajectories over time, with TRA values categorized into four groups. The upper panel indicates individual-level parasite exposure over time, classified as parasite-negative visits (asterisks), visits with asymptomatic parasitaemia (both microscopic and sub-microscopic, white dots), and clinical malaria episodes (black dots). Sample collection dates are indicated on the x-axis.

For blocker 1 (female; 7 years of age in 2013) who acquired high levels of TRA (≥80%) over time, most antibody densities similarly increased over time with the exception of the average response to TRA-associated hits on the microarray that remained low (Figure 7A). Interestingly, transmission reducing activity developed following a year with six time-points when asymptomatic parasitaemia was detected. Blocker 1’s matched control (male; 7 years of age) had numerous episodes of clinical malaria, with sexual-stage antibody densities against Pfs230-CMB, Pfs48/45, and PfMDV1 and TRA peaking (at 73% TRA) during a visit when asymptomatic parasitaemia was detected. After this visit the frequency of infections decreased significantly, and both TRA and sexual-stage antibody densities decreased also. For blocker 2 (female; 33 years of age), sexual antibody responses were initially high (almost all in the upper quartile), matching a period of high TRA (≥80%). TRA and antibody levels subsequently decreased over a period with negligible parasite exposure (one moment of sub-microscopic parasite carriage during a 31-month period with 21 sampling time-points; Figure 7B). Surprisingly, blocker 2’s control (female; 38 years of age) showed high and persisting levels of sexual-stage antibody responses without functional activity. Blocker 3 (female; 55 years of age) showed consistent high levels of TRA (≥80%); antibody densities (other than Pfs230-CMB, PfMDV1, and Pfs47-Del2) remained in the upper two quartiles for the entire period of observation. The follow-up of this consistent blocker was also characterised by long periods of asymptomatic parasitaemia followed by (shorter) periods of parasite negative visits. Blocker 3’s control (female; 46 years of age) also had visits with asymptomatic parasitaemia, however the antibody densities remained in the lowest two quartiles for most of their follow-up (Figure 7C). When combining all blocking samples (n=25) from these three donors, all had detectable antibodies against Pfs230-CMB and Pfs48/45, though Pfs230-CMB levels were sometimes low (Supplementary Figure 5). Amongst the 14 blocking samples with antibody densities in the lowest two quartiles for Pfs230-CMB, Pfs48/45, or both, 13 (92.9%) were in the top quartile (≥75%) of antibody responses against PF3D7_1016300, followed by PF3D7_1120600 (85.7% in the top quartile, n/N=12/14) and PF3D7_0608600 (78.6% in the top quartile, n/N=11/14, Supplementary Figure 6). In comparison, only two of these samples had anti-Pf77 or anti-PfMDV1 antibody densities in the top quartile (14.3%, n/N=2/14) and a single sample had anti-PfHAP2-D2 densities in the highest quartile (7.14%, n/N=1/14). Few samples with TRA < 80% (low to intermediate TRA) had antibody levels in the top quartile for the three array targets (7.0%, n/N=4/57, for PF3D7_1016300 and PF3D7_1120600; 12.3%, n/N=7/57, for PF3D7_0608600), whereas this proportion was highest for antibodies against Pf3D7_1449000 (31.6%, n/N=18/57), PF3D7_1245100 (29.8%, n/N=17/57), and Pfs47 (26.3%, n/N=15/57).

## Discussion

In the present study, we assessed antibody responses to gametocyte-expressed antigens and their association with functional transmission reducing activity (TRA) in a longitudinal cohort study of individuals naturally exposed to *P. falciparum*. We observed high-levels of functional TRA in 1.6% of individuals tested; an even smaller subset of individuals had persisting high-levels of TRA. Naturally acquired antibody responses against sexual-stage antigens were associated with high-level TRA, including antibodies against Pfs48/45, Pfs230, PfMDV1, PfHAP2, Pf77, Pfs47, and nine gametocyte-expressed antigens that were identified using a protein micro-array.

Sexual-stage immunity contributes to the variation in onward transmission potential. Prior to the current study, research on the sero-epidemiology of sexual-stage immunity was almost exclusively restricted to microscopy-positive gametocyte carriers, and demonstrated a causal role for anti-Pfs48/45 and anti-Pfs230 antibodies in mediating functional TRA [20]. The prevalence and longevity of functional TRA in naturally exposed populations remained understudied, as is the role of (recent) parasite exposure in the acquisition and maintenance of TRA. Moreover, several recently identified gametocyte antigens (PfMDV1, PfHAP2, Pf77, Pfs47) were shown to induce functional antibodies in rodent studies [33–36], but were not yet examined in relation to naturally acquired TRA.

Our cross-sectional data from 313 naturally-exposed Ugandan individuals reveals that immune responses to recently identified gametocyte-specific antigens are associated with high-level TRA and increase with age. This is in line with earlier findings on antibodies against the well-known gametocyte-antigens Pfs48/45 and Pfs230 [19, 45, 46] but contradicts an earlier hypothesis that gametocyte immunity is most prevalent in younger age groups that experience higher gametocyte loads upon infection [32, 47]. Uniquely, we provide strong indications that asymptomatic parasite carriage is associated with the acquisition and persistence of gametocyte immunity. Gametocyte antibody responses were positively associated with asymptomatic parasite carriage, as was strong functional TRA. Findings from a comparable Ugandan cohort [2] and other settings [48, 49] demonstrated that asymptomatic infections can be long-lasting and harbour long phases of (low-density) gametocytes that may drive the higher sexual-stage responses we observe. Moreover, we observe that established markers of recent asexual parasite infection better predict high-level TRA than markers of cumulative parasite exposure. This supports earlier hypotheses that gametocyte immunity is comparatively short-lived [19]. In line with this, we find that gametocyte-specific antibody responses are generally lost over time with kinetics that resemble markers of recent infection exposure.

We provide evidence that intermediate levels of TRA are not just an artefact of the biological assays used to assess them. Prior studies have set a threshold for functional TRA (≥80%) based on its reproducibility [43, 50], while it was acknowledged that lower levels of TRA can also be highly relevant [51]. We present evidence for the existence of intermediate levels of TRA (50–79%) that is i) associated with higher antibody recognition of activated female gametes, with a trend towards higher Pfs48/45 and Pfs230 levels, and ii) commonly observed in longitudinal samples of cohort participants who blocked transmission at other time-points. The impacts of intermediate versus high-level TRA on transmission under natural conditions remains unknown but the fact that 7% of individuals in our cross-sectional survey exhibit TRA ≥50%, suggests potential epidemiological importance.

Using longitudinally collected samples, we demonstrate a level of consistency in functional TRA. Individuals with high levels of functional TRA in our primary cross-sectional survey were more likely to show functional TRA at other time-points. In contrast, an earlier study examining longitudinal patterns in gametocyte immunity found no evidence for persistence of functional TRA; this study used a slightly higher threshold for defining functional TRA (90%) [32]. We observed one individual with high levels of functional immunity that persisted over several months of non-exposure. Although we may have missed very short-lived infections [52] in our monthly surveys, our findings are in line with persisting Pfs48/45 and Pfs230 antibodies despite absence of parasite exposure in in Ghana [53, 54] and the Gambia [21].

Only 3.6% of samples with high-level TRA was completely seronegative for both Pfs48/45 and Pfs230 antibodies. A previous multi-country study ((Burkina Faso, Cameroon, The Gambia and Dutch migrants) reported that ~45% of high-level TRA samples were seronegative for both antigens [20]. Our immunoassays used full-length Pfs48/45 (448 amino acids, domains 1–3), whereas the previous study measured antibodies to only domains 2–3 [20]. Even though domains 1 and 3 are considered the primary targets of potent transmission-reducing antibodies [23, 55], the high seropositivity observed in this study may, at least in part, be driven by antibody responses to the immunogenic yet non-transmission blocking domains 2 [55, 56]. Both the previous and the present work quantified antibodies against Pfs230-CMB, a construct comprising the pro-domain and domain 1, previously shown to be the target of transmission blocking antibodies [57–59]. Pfs230 is a large protein (3135 amino acids, divided into 14 domains), and recent work indicates that functional antibodies develop against additional regions – such as domain 12, [60], domain 7 [60, 61], domain 4 [14] and domains 13–14 [15]. Future work is needed to characterize antibody responses to these domains and their relationship with TRA in naturally infected individuals. Previous work also showed that not all antibodies against domain 1 of Pfs230 are functional [58] and that functionality can be epitope specific. Nevertheless, while domain (and epitope) specificity are essential to antibody functionality, antigen-specific TRA may be rare [20] and naturally occurring functional TRA could predominantly be driven by combinations of (potentially low-level) sexual-stage antibody titres.

Our work reinforces the notion that antigens other than Pfs48/45 and Pfs230 are likely to be relevant for high-level TRA. Antibodies against PfMDV1, PfHAP2, Pf77 and Pfs47 were associated with TRA. While antibodies against PfMDV1, PfHAP2, Pf77 and Pfs47 are detectable in most samples with functional TRA, we observed no consistent immune signature of TRA and different (combinations of) antibody responses are observed for different samples and individuals. Using a protein array comprising 588 *P. falciparum* antigens, we identified nine targets associated with functional TRA. Among these, PF3D7_1038400 (Pf11-1) and PF3D7_1449000 (PfGEST) had previously been identified as TRA-associated [20]. In subsequent immunization experiments, functionality could not be confirmed for PfGEST expressed in a wheat germ cell free system, possibly due to improper folding [62] or because PfGEST is not a direct target of functional transmission-blocking antibodies. Monoclonal antibodies against a product of *Pf11.1* were previously reported to reduce oocyst formation [63]; in our own immunisation experiments a fragment of Pf11-1 induced antibodies with moderate TRA in mice but not rats [64]. Pf11-1 contains a glutamate-rich repeat region that can induce cross-reactive antibodies with similar regions in Pfs230 [65]. In the current study, antibodies to Pfs230 were measured using a construct (aa 444–730) outside its glutamate rich repeat region (aa 381– 441), making cross-reactivity unlikely, although responses against Pf11-1 may still reflect cross-reactivity with other antigenic glutamate rich-repeat regions. Restricting our analysis to blocking samples without high Pfs48/45 and Pfs230 antibody responses, three array-identified targets showed relatively high antibody levels; PF3D7_1016300, PF3D7_1120600, and PF3D7_0608600. PF3D7_0608600 encodes a protein of unknown function; glycophorin binding protein (GPB130, PF3D7_1016300) is involved in red blood cell adhesion and rigidity [66]; and the *P. berghei* ortholog of PF3D7_1120600 (a DDRGK domain containing protein) is essential for in gametocyte formation [67]. To our knowledge, these antigens have not been studied in relation to antibody-mediated TRA, and future work is warranted to assess their causal role and transmission-blocking potential.

Our study has several limitations. Assessment of transmission blocking activity is resource intensive – and whilst our finding that high-level TRA is rare is a relevant finding, small sample sizes will have affected our power to detect associated antibody responses. A further limitation is that gametocyte exposure was not systematically assessed, and given that gametocyte densities fluctuate over the course of infections [68], future studies would benefit from directly evaluating the relationship between gametocyte exposure and sexual-stage antibody responses. Nevertheless, it was repeatedly demonstrated that in asymptomatic infections, asexual parasite density is positively associated with gametocyte density [2, 3]. We thus feel confident that the associations we present between TRA and preceding asymptomatic infections broadly reflect gametocyte exposure. Limitations were also present in our serological methods; microarray design timelines did not allow inclusion of Pf77, and the array’s Escherichia coli-based cell-free expression system may have limited natural protein folding. For all functional and serological assays we focused on IgG-mediated functionality, as this is the only isotype studied in relation to natural TRA to date, although IgM responses to sexual-stage antigens also occur naturally [69] and should be examined alongside IgG in future studies of natural settings.

To conclude, our study shows that naturally occurring functional TRA may persist over time and is related to sexual-stage antibodies and parasite exposure. Exploring the functionality of novel sexual-stage antibodies and assessing on the lesser known natural kinetics of well-studied sexual-stage antibodies, our data further improves the understanding of naturally occurring *P. falciparum* transmission reduction. In so doing, it may inform the development of current and future transmission-blocking vaccine candidates, and inform the evaluation of such interventions in whole populations.

## Methods

### Study site

A prospective cohort study was conducted between October 2011 and September 2017 in Nagongera subcounty in Tororo District, Eastern Uganda. In the years 2011 and 2012, malaria transmission in Tororo was high with an estimated inoculation rate (EIR) of 310 infective bites per person per year [39, 41]. Indoor residual spraying (IRS) was first introduced in December 2014-January 2015 followed by rounds in June-July 2015 and in November-December 2015. In 2015, the first year after implementation of IRS, the annual EIR dropped to 12.4 [41].

### Study procedures

Screening and enrolment of households and study participants has been described in detail elsewhere [39, 70]. Briefly, 100 households were initially selected between August and September 2011. To replace households that dropped out, additional households (n=7) were enrolled between August and October 2013. From selected households, one primary caregiver aged 18 years or older and children were eligible if they were between 6 months and 10 years of age and met the additional enrolment criteria [39, 71]. All eligible participants or parents of participants (for those <10 years of age) provided written informed consent prior to enrolment. The cohort was dynamic, study participants that had reached 11 years of age were excluded and all newly eligible household members were enrolled. Children were invited to the study clinic for scheduled visits once every three months until November 2014, then monthly from December 2014. Adults had scheduled visits every three months until July 2016, then monthly from August 2016 onwards. Routine evaluation during scheduled visits consisted of temperature recording and thick blood smear reading. A blood smear was considered negative when microscopic examination of 100 high-power fields did not reveal asexual parasites. When blood smear negative, presence of sub microscopic parasite densities was assessed using loop-mediated isothermal amplification (LAMP) [72]. Cohort participants received medical care free of charge at a designated study clinic in Nagongera that was open 7 days a week. A blood smear was obtained by finger prick from participants who presented at the clinic with a fever (tympanic temperature ≥38°C) or history of fever in the past 24 hours; malaria episodes detected during scheduled or unscheduled visits were treated with artemether-lumefantrine according to national guidelines. For serological assessments, plasma was collected from finger prick or venipuncture blood samples during routine visits.

### Sample selection

As part of a large, untargeted screen to identify samples with high levels of transmission reducing immunity for the identification of naturally acquired transmission blocking monoclonal antibodies [55, 58], 1496 plasma samples collected between March 2013 and September 2017 from 394 individuals were screened against gametocyte lysate [55]. These samples formed the starting point for the current analysis. To allow for an unbiased, comprehensive assessment of sexual-stage immune responses in the cohort while retaining all available information from prior work, we supplemented the initial selection with additional samples to ensure at least one sample was assayed for each cohort participant contributing blood samples to the study in the March-October period (spanning both transmission seasons) for each year between 2013–2017. This resulted in a total of 2532 samples from 433 individuals (38.1% had observations for all 5 years, 20.6% for four years, 14.1% for three years, 11.8% for two years, and 15.4% for one year only).

To determine the prevalence of functional transmission blockade, we selected a single sample per individual based on plasma availability within a broad time-window (March 2013 and July 2014), resulting in a selection for SMFA of 313 samples/individuals. In addition to this cross-sectional selection, we aimed to study the dynamics of (functional) immune responses over time. For this, we included additional follow-up samples for a selection of individuals. From individuals who blocked transmission (TRA ≥80%) in the first SMFA, follow-up samples were selected for further rounds of SMFA, with blocking samples repeated at least twice to confirm blockade (mean TRA ≥80%). Additionally, a group of ‘control’ individuals was selected who did not block transmission in the cross-section, based on household proximity and similarity in age to blocker individuals. Follow-up samples from control individuals were included in SMFA. Control individuals that blocked transmission later in their follow-up were categorised as individuals who blocked transmission. Addition of the longitudinal sample selection from blockers and controls resulted in a final selection of 611 samples from 313 individuals included in SMFA (including all cross-sectional samples). Some of the samples selected as part of the longitudinal study (n=68 samples) were not in the initial sample set of 2532 samples. The addition of these samples resulted in a grand total of 2600 plasma samples from 433 individuals.

The grand total of 2600 plasma samples from 433 individuals was used for assessing antibody kinetics and prevalence in relation to parasite exposure, and antibodies against Pfs48/45, Pfs230-CMB and gametocyte lysate were measured in these samples using ELISA. Sexual-stage antibody responses were compared to a panel of asexual-stage antibody responses measured using Luminex. Antibodies against PfHAP2-D2, PfMDV-1, Pfs47-Del2, and Pf77 were measured by ELISA in all samples included in SMFA (n=611 samples, 313 individuals) to assess the functionality of naturally occurring antibodies against these sexual-stage antigens.

### ELISA

Antibody responses against gametocyte extracts were assessed by enzyme linked immunosorbent assay (ELISA) as previously described [73]. Briefly, 100uL of *P. falciparum* NF54 gametocyte extract, corresponding to 75,000 gametocytes, was added to each well on a Nunc MaxoSorp 96-well plate (ThermoFisher) and incubated overnight. Plates were blocked with 150 μL of 5% milk/PBS, plasma samples were diluted in 1% milk/PBS (1:5,4000) and tested in duplicate. 100 μL of diluted (1:40,000 in 1% milk/PBS) horseradish peroxidase (HRP)-conjugated Goat anti-Human-IgG antibody (31412, Invitrogen) was added to each well. HRP activity was detected using 100 μL of tetramethylbenzidine (TMB); the optical density (OD) was read at 450nm on an iMark^™^ microplate absorbance reader (Bio-Rad). Antibodies against specific antigens were quantified in separate ELISAs. Plates were coated overnight (at 4°C) with 100 μL of recombinant full length Pfs48/45 (0.5 μg/ml), Pfs230CMB (aa 444–730 of Pfs230, 0.5 μg/ml) [57], Pfs47-Del2 (aa 178–229 of Pfs47, 1 μg/ml) [36, 74], PfHAP2-D2 (aa 176–195 of PfHap2, 1 μg/ml) [37], recombinant Pf77 (aa 315–644, 0.5 μg/ml) [33] or recombinant PfMDV-1 (aa 30–221, 0.25 μg/ml) [33]. All ELISA plates were blocked with 150 μL of 5% milk/PBS for 1 hour plasma samples were diluted in 1% milk/PBS (1:200, or serial dilutions of 1:200 to 1:800 for high responders) and tested in duplicate. The subsequent ELISA steps were performed as described above. To normalise optical densities (arbitrary units), a standard curve (7 serial dilutions in duplicate starting at 1:100) of highly reactive human sera was used (serum from a Dutch missionary for Pfs48/45, Pfs230CMB and gametocyte lysate, and pooled sera from Tanzanian gametocyte carriers for Pfs47-Del2, PfHAP2-D2, Pf77, and PfMDV-1) and analysed in ADAMSEL FPL (http://www.malariaresearch.eu/content/software).

### Luminex

Antibody responses against a panel of recombinant blood-stage P. falciparum antigens, circumsporozoite protein (CSP), and Anopheles gambiae salivary gland protein 6 (gSG6) were measured using a multiplex Luminex bead assay (Supplementary Table 5). Tetanus toxoid was included as a positive vaccine control, and glutathione S-transferase (GST) served as a negative control for expression-tag cross-reactivity. Serum samples were diluted 1:400 in buffer B (1xPBS, 0.05% Tween, 0.5% BSA, 0.02% sodium azide, 0.1% casein, 0.5% polyvinyl alcohol (PVA), 0.5% polyvinyl pyrrolidone (PVP), E.coli lysate 15.25ug/mL) and incubated for one day prior to screening. Antigen-MagPlex© COOH-bead coupling optimisation’ large-scale couplings’ and Luminex IgG detection multiplex bead assays (MBA) were performed as described previously [75]. Briefly, 50μL of antigen-coupled beads (approximately 1000 beads per analyte per well) were incubated with 50μL of diluted serum or control samples for 90 minutes. Beads were washed and incubated with 50μL of anti-human IgG Fcγ secondary antibody (109-116-098, Jackson ImmunoResearch; 1:200 dilution) for 90 minutes. Following additional washes, beads were incubated in 50μL of buffer A (1xPBS, 0.05% Tween-20, 0.5% BSA, 0.02% sodium azide) for 30 minutes, washed, and resuspended in 100μL of PBS. Blank-adjusted median fluorescence intensity (MFI) values were acquired using a MAGPIX system.

Quality control samples included serially diluted hyperimmune Tanzanian sera (CP3), the WHO international reference standard for *P. falciparum* (10/198, NIBSC), hyperimmune Ugandan sera (PRISM), malaria-naïve European sera (Public Health England, 2016), and buffer-only blanks. Plates with positive control values exceeding two standard deviations from the mean in Levey-Jennings plots were repeated. CP3 curves were used to adjust for inter-plate variability using LOESS normalisation.

### SIFA

A surface immuno-fluorescence assay (SIFA) was used to detect antibody reactivity against live female *Plasmodium falciparum* gametes. 16-day old N-acetyl glucosamine treated NF54 gametocyte cultures were collected and centrifuged (2,000g at RT, 10 min). Gametocytes were allowed to activate on a roller bank for 45 minutes at RT in the presence of FCS, upon which they were centrifuged (2,000g at 4°C, 10 min). The gamete pellet was resuspended in FCS and loaded onto a 7mL layer of 11% w/v Accudenz (Accurate Chemical). After centrifugation (7,000g at 4°C, 30 min, no brake), female gametes residing in the top layer were harvested and counted using a Bürker-Turk counting chamber. Gametes were then prepared for microscopy by washing with PBS supplemented with 0.5% FCS and 0.05% sodium azide. Serum samples were diluted (1:8,000) in the same PBS/FCS buffer and added to gamete preparations (1:1). Hoechst was added at dilutions of 1:100 to stain gametes and secondary antibody Alexa Fluor TM 488 goat anti-human IgG (H+L) was added at dilutions 1:200. All gamete preparations were incubated with secondary antibody at 4°C for 30 min. Antibody reactivity against female gametes was calculated as the number of labelled gametes divided by the total number of gametes in the well (i.e. the percentage of positive labelled gametes).

### Micro-array

Protein microarrays were designed at LSHTM, Radboud University, and UC Irvine, and printed by Antigen Discovery Inc. (Irvine, California, US). Protein selection was based on a gametocyte-specificity analysis using detection frequencies across 11 proteomic datasets, described in detail elsewhere [76]. Briefly, proteins were binned by abundance and weighted according to retrieval rates in two curated “gold-standard” gene sets—45 asexual and 41 gametocyte-specific genes. High retrieval of gametocyte markers and absence of asexual markers yielded a high gametocyte score, calculated as the fraction of retrieved gametocyte over non-gametocyte genes. Scores were log-transformed and summed across datasets.

Additional inclusion criteria (Supplementary Table 3) prioritised proteins likely to be expressed on gamete surfaces (gene ontology, domain prediction, or empirical evidence) or previously associated with gametocyte exposure [77], transmission-blocking immunity [20], or antibody recognition of immature giRBC surfaces [78].

A total of 604 *P. falciparum* genes were selected for cloning from the 3D7 reference genome; sequences >1000 amino acids were split into overlapping fragments. PCR and cloning succeeded for 588 genes (1135 sequences). All were expressed using an IVTT system (5 Prime, Gaithersburg, MD, USA) following the manufacturer’s protocol and described elsewhere [79, 80].

Arrays were printed on 8-pad nitrocellulose-coated AVID slides using an Omni Grid Accent robotic printer. Each (sub)array included IgG positive controls and negative IVTT-only controls for normalisation. Samples were processed as previously described, with minor modifications: serum dilution was 1:200 and secondary antibody (Southern Biotech, Goat Anti-Human IgG-TXRD) was used at 0.5 μg/mL [20, 79]. Arrays were scanned using a GenePix 4300A scanner. Local background was automatically assessed, and foreground MFI calculated using irregular threshold pixel density mapping. Background correction used the backgroundCorrect function from limma [81]. Corrected values were log2-transformed and normalised by subtracting the median of the negative IVTT controls within each set of four subarrays. Final values are log2 MFI ratios relative to background reactivity (0 = background level; 1 = two-fold above background).

### Standard membrane feeding assays

IgGs were purified from selected plasma samples as described elsewhere [20] using Ab SpinTrap^™^ columns (Cytiva) and concentrated to the original plasma volume in milli-Q using Vivaspin 20 centrifugal concentration columns (Sartorius AG, Goettingen, Germany). IgG-mediated transmission-reducing activity (TRA) was assessed in standard membrane feeding assays (SMFA) using 3–5-day-old female *Anopheles stephensi* mosquitoes (Sind-Kasur Nijmegen strain). Mosquitoes were maintained at 30°C, 70–80% humidity under a 12-hour light/dark cycle.

Either NF54 [82] or transgenic NF54HT-GFP-Luc [83] mature *Plasmodium falciparum* gametocytes were prepared with packed red blood cells as described previously [42, 83]. For each feeding experiment, 90 μL of purified IgGs, 90 μL of FCS, and 35 μL of human malaria-naïve serum containing active complement were added to a gametocyte/packed red blood cell mixture to a final volume of 270 μL and kept at 37 °C before mosquito feeding. Two control groups were included per assay and included a feed with a mixture of 90 μL FCS, 90 μL milliQ and the same gametocyte/red blood cell mixture (270 μL final volume per feed).

Mosquito infection status was assessed differently for the two parasite strains as described previously [20]. For NF54, 20 mosquitoes were dissected 6–8 days post feeding and stained in 1% mercurochrome upon which oocysts were counted by expert microscopists. For NF54HT-GFP-Luc, mosquitoes were killed on day 8 post feeding by freezing, and 20 mosquitoes were homogenised in two pools of 10. Mosquito homogenates were well mixed and assayed for bioluminescence by lysis an by addition of luciferase assay substrate using a Luciferase assay kit (Luciferase assay system (E1501), Promega, USA).

TRA of plasma samples was defined relative to the control groups in every SMFA. For NF54 assays, TRA was calculated as the percentage reduction in oocyst density (oocyst count per mosquito midgut) between test and control feeds. For NF54HT-GFP-luc assays, TRA was calculated as the average percent difference in luminescence intensity between test and control mosquito pools as described previously [42]. Samples with TRA ≥ 80% were repeated to ensure robustness of the phenotype. For repeated assays, the mean TRA was used as the final value.

### Statistical analyses

Parasite prevalence was calculated as the number of routine visits with microscopic or sub microscopic parasites divided by the total number of parasite assessments. Malaria incidence was calculated using all cohort data by counting the number of new episodes (at least 14 days apart) and dividing it by the total person years of observation. Parasite incidence was calculated similarly except parasite positive visits without symptoms were also counted. Pfs230CMB and full-length Pfs48/45 were included in both Luminex and ELISA and comparison of fluorescence with normalised OD values yielded a correlation co-efficient of 0.51 (P < 0.001) and 0.46 (P < 0.001), respectively (Spearman’s rank). Mean OD values from ELISA were categorized as seropositive when they were at least three standard deviations above the mean OD value from 8 independent malaria-naïve individuals included on every assay plate. For Luminex data on antibody densities against asexual antigens, plasma samples were labelled positive when the fluorescence signal was at least three standard deviations above the mean of independent negative controls included on every assay plate. This method was chosen instead of a mixture model considering the potential challenge of identifying a negative population for *P. falciparum* antigen exposure in an area of historically high malaria transmission [84].

Seroprevalence was calculated as the number of seropositive routine visits divided by the total number of routine visits with seropositivity data. Generalized linear models (GLM) with a binomial distribution and logit-link were used to examine age-trends in antibody seropositivity. Models were run separately for different antibody responses using data from the 2013–2014 cross-section (single-time point data). Participant age (integer) was included as a predictor variable. All available serological data was used for the comparison of seropositivity before (2013–2014) versus after (2016–2017) implementation of IRS, and individuals were categorized in the following age-categories: below three years, 3–11 years and 18 years or older. To account for within-individual dependencies resulting from repeated measures, generalized estimating equation (GEE) models were used with an exchangeable correlation structure for all age categories separately with seropositivity as outcome variable and time-frame (pre versus post-IRS) as a predictor variable. Within each experimental outcome, p-values were adjusted using the Benjamini Hochberg approach for multiple testing.

Generalized linear models with a binomial distribution and logit-link were used to examine the relationship between antibody seropositivity and history of parasite exposure. Models were run separately for the different antibody responses and for different exposure time-frames (past 3 months, past 12 months). Model outcome was set as the seropositivity status for a given antibody response at timepoint X. For asymptomatic infections, parasite exposure was calculated per participant as the number of visits with sub microscopic parasitaemia or microscopic parasitaemia, expressed as proportion of the total number of scheduled visits for that participant and time-frame (i.e. a proportion ranging from 0–1). Clinical incidence was calculated as the sum of new clinical episodes (separated by at least 14 days) divided by the person-month of observation for a given participant and time-frame. Observations were excluded if they had less than 2 or 4 informative visits (or less than 2 or 6 person-months of observation) in the past 3 or 12 months, respectively. The exposure covariates were included as separate variables in every model and all models accounted for participant age (in categories) and whether observations occurred during or before implementation of IRS. To account for repeated measures, a random person-effect was included in every model. P-values were adjusted for multiple testing within each experimental outcome using the Benjamini Hochberg approach.

Relative change in antibody density (*y*) since the last parasite positive visit was calculated per antibody type using the following formula, where *z* is the antibody density at the last parasite positive visit and *x* the current antibody density:

$$y=\frac{x}{z}*100$$

Observations were excluded if the current antibody density occurred at a parasite positive visit, since this was considered as a new infection.

Antibody responses were used to predict transmission blockade in observations from the cross-section (single time-point analysis) using receiver operating characteristic (ROC) curves. The area-under-the-curve (AUC) of ROCs was calculated using the pROC package. Two ROCs were compared using a DeLong test. To account for within-individual dependencies resulting from repeated measures, generalized estimating equation (GEE) models were used with an exchangeable correlation structure to compare antibody densities between the three TRA categories (−50 to 9% TRA, 10 to 79% TRA, and ≥80% TRA). Within each experimental outcome, p-values were adjusted using the Benjamini Hochberg approach for multiple testing.

To be able to visualize antibody responses and compare antibody responses from ELISA with those from the micro-array, we grouped antibody densities into quartiles which allows scale independent comparisons. Initially, signal intensities from the 10 micro-array targets were averaged and grouped into quartiles to allow visualization of the overall array response. In subsequent analyses, signal intensities were categorized by protein, averaging only signal intensities from fragments of the same protein.

All analyses were performed in R (version 4.4.1, 2024-06-14). All data from the underlying cohort study are publicly available on ClinEpiDB (PRISM ICEMR Cohort), and all data used in the present manuscript have been made available through Figshare.

## Supplementary Material

1

## Figures and Tables

**Table 1.** General characteristics of individuals from the 2013–2014 cross-section. Samples from the 2013–2014 cross-section (one sample per individual) were selected for standard membrane feeding assays in which the transmission reducing activity (TRA) was determined and categorized into low (−50% - 9%), intermediate (10–79%) and high TRA (≥80%). Samples with TRA < −50% were excluded due to a potential effect of transmission enhancing samples (n=13 samples), resulting in 300 samples from 300 individuals.

	TRA category		
	Low	Intermediate	High
Total (N)	130	165	5
Age in years (median, IQR)	7 (5–29)	7 (8–10)	33 (8–54)
Parasite prevalence by LAMP (%, n/N)	30.0% (39/130)	31.2% (52/165)	60.0% (3/5)
Parasite prevalence by microscopy (%, n/N)	31.5% (41/130)	30.3% (50/165)	40% (2/5)
Clinical malaria (%, n/N)	3.8% (5/130)	5.4% (9/165)	0% (0/0)

**Table 2.** Transmission reducing activity in relation to historical parasite exposure. Transmission reducing activity was determined in standard membrane feeding assays and categorized into low (−50% - 9%), intermediate (10–79%) and high, or functional, TRA (≥80%); samples with a TRA < −50% were excluded. For every observation with a TRA value above −50%, the proportion of past parasite positive visits was calculated. This was done for visits with asymptomatic (microscopic and sub microscopic) parasitaemia and clinical malaria episodes separately.

	TRA category		
	Low	Intermediate	High
Total past visits with parasite assessments (N)	3457	6439	606
**Proportion of past parasite positive visits (%, n/N)**			
Asymptomatic sub microscopic parasitaemia	9.5 (330/3457)	12.0 (772/6439)	16.3 (99/606)
Asymptomatic microscopic parasitaemia	24.6 (851/3457)	27.8 (1791/6439)	42.9 (260/606)
Clinical malaria	31.6 (1091/3457)	28.6 (1841/6439)	16.3 (99/606)
