# Supplementary material for: The kinetics and transmission-blocking dynamics of P. falciparum sexual-stage antibody responses in a six-year cohort of Ugandan children and adults
Source: Res Sq. 2026 Feb 9:rs.3.rs-8733488. Preprint. [Version 1] doi: 10.21203/rs.3.rs-8733488/v1 (PMC12919170; doi:10.21203/rs.3.rs-8733488/v1)
Supplement: 1 [file NIHPPRS8733488V1-supplement-1.pdf]

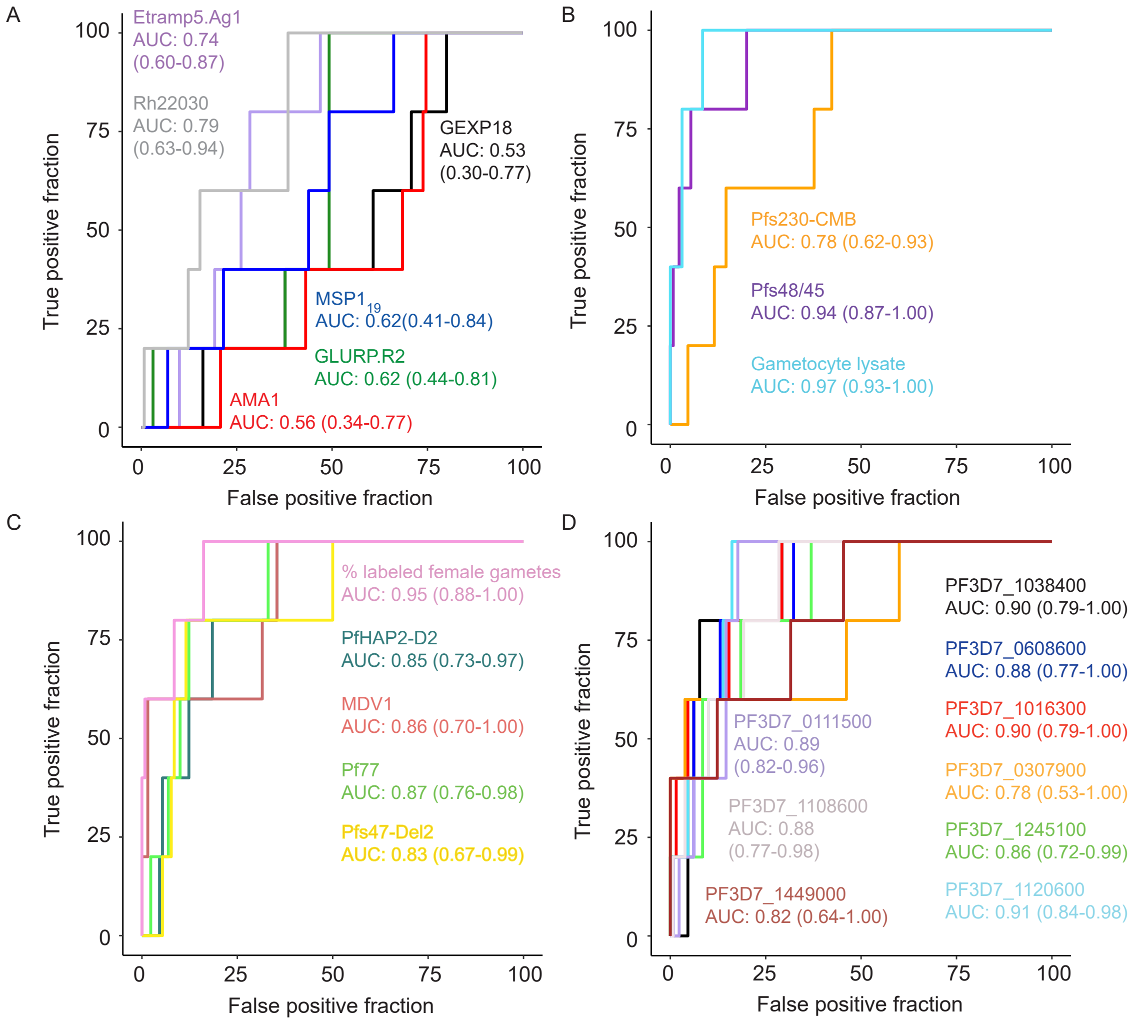

Supplementary Figure 1. Antibody levels against sexual-stage *P. falciparum* antigens predict malaria transmission blockade. Receiver operating characteristic (ROC) plots showing the performance of asexual (a,b) and sexual-stage (c,d) antibody densities in classifying functional transmission reducing activity. A threshold of 80% reduction in oocyst density was set as functional TRA (i.e. transmission blockade) and tested against samples with low-to-intermediate-level TRA (-50% - 79% TRA). The lower limit of TRA was set to exclude a potential effect of transmission enhancing samples. Samples from a 2013-2014 cross-sectional dataset were used to avoid bias from correlated measurements within individuals and we only used samples with complete antibody data so that all ROC plots are based on the same dataset (n=130 samples with -50%-9% TRA, n=5 samples with ≥80% TRA). Area under the curve (AUC), together with the 95% confidence intervals (CI) are indicated in text for each antibody response separately.

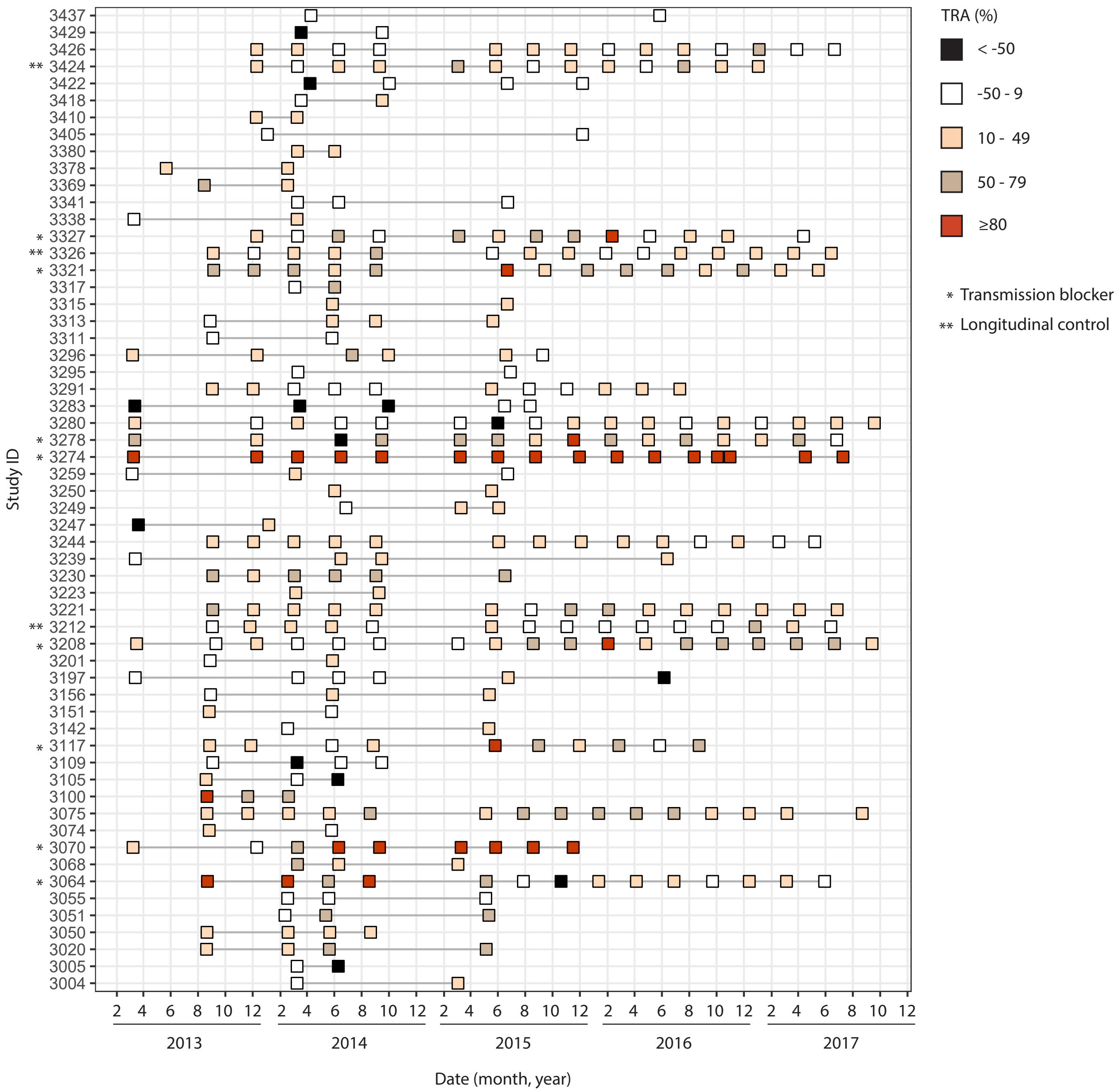

Supplementary Figure 2. Longitudinal follow-up with measurements of transmission reducing activity (TRA). Line plot depicting individual TRA trajectories over time for individuals with at least two TRA measurements. Sample collection dates are depicted on the x-axis, and TRA categories are depicted in coloured squares for each visit. Individual IDs are depicted on the y-axis and asterisks indicate individuals that are selected as longitudinal blockers (*), individuals who had functional TRA (≥80%) at least once and longitudinal controls (**), individuals who never had functional TRA and were age- and household-matched to a longitudinal blocker.

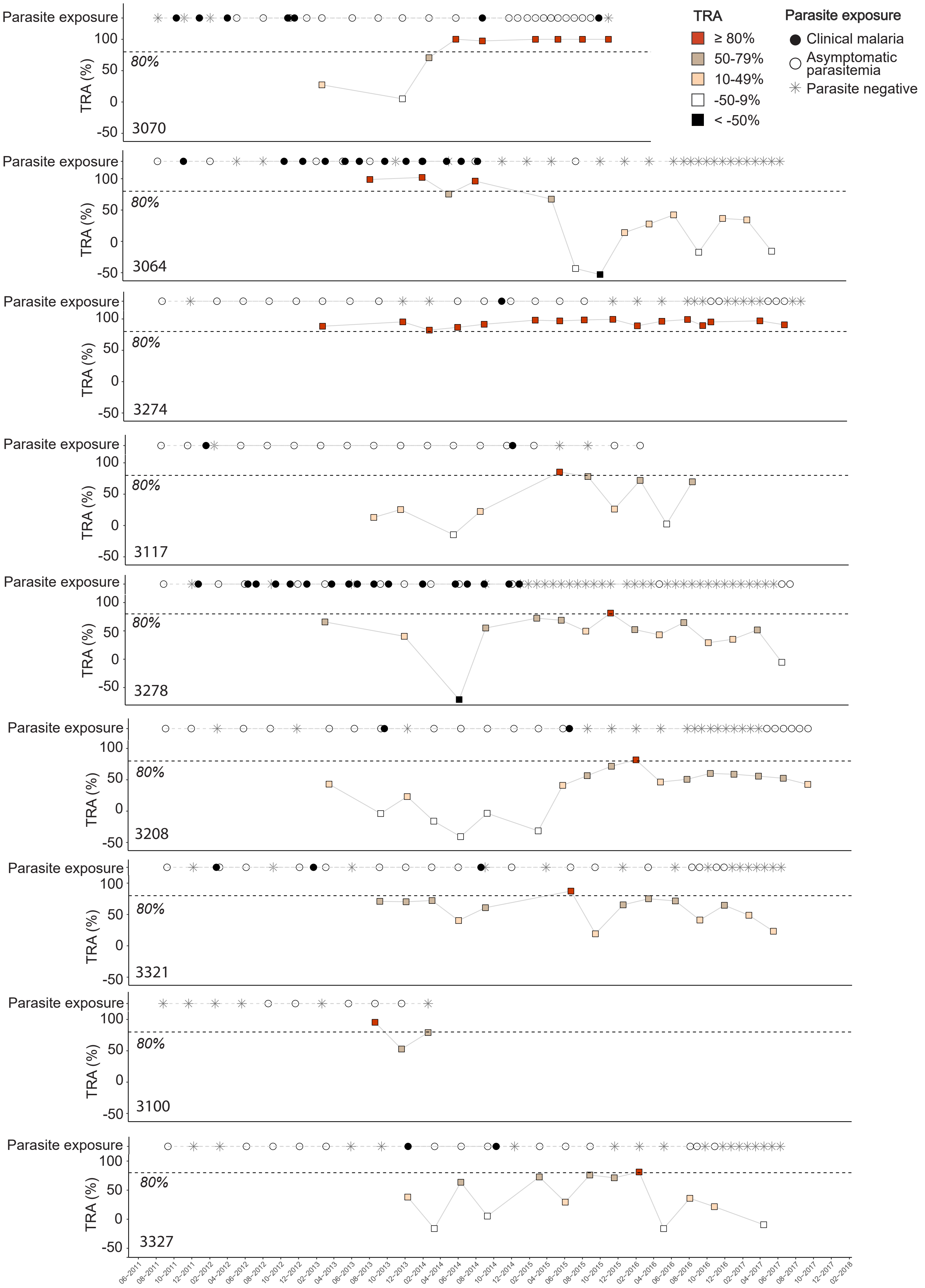

Supplementary Figure 3. Parasite exposure and transmission reducing activity over time. Line plots depicting trajectories of TRA and parasite exposure over time for nine individuals that had at least two TRA observations and at least one observation with functional TRA (≥80%). The upper panel depicts individual-level parasite exposure over time, classified as parasite-negative visits (asterisks), visits with asymptomatic parasitaemia (both microscopic and sub-microscopic, white dots), and clinical malaria episodes (black dots). TRA categories are depicted in the lower panel by coloured squares. Individual IDs are indicated in text in the lower panel. Sample collection dates are indicated on the x-axis.

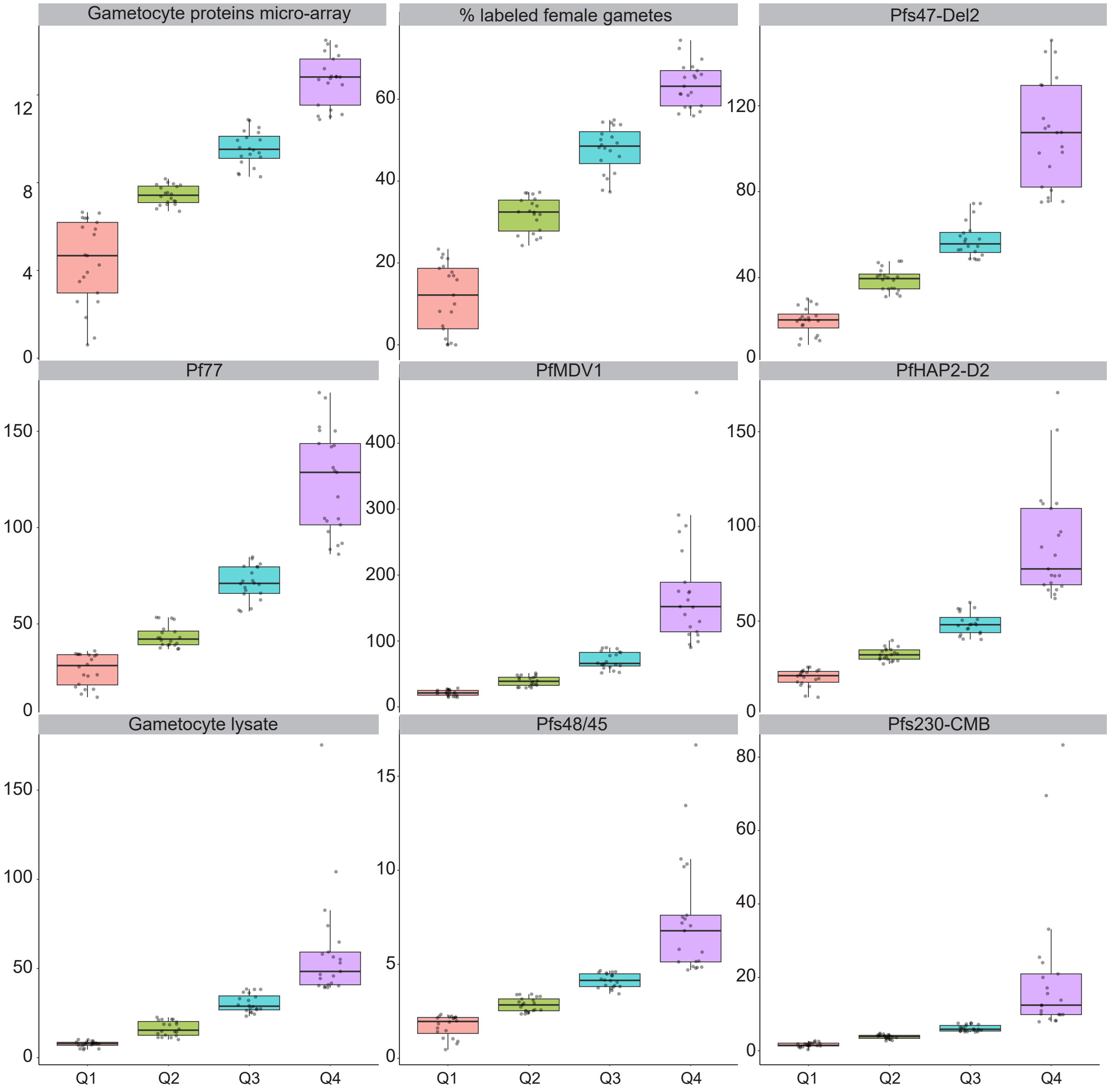

Supplementary Figure 4. Box plots depicting antibody levels in quartiles. To avoid bias from fluctuations in serologic metrics across assay types, specific antibody densities and array responses (averaged for the 10 hits associated with transmission reducing activity [TRA]) were dived into quartiles (using the 25th, 50th, 75th percentile). Quartiles were computed using all observations from three individuals with at least one observation with functional TRA (≥80%, i.e. blockers), and all observations from 3 control individuals that never achieved functional TRA and were age- and household-matched to the blockers (n=82 samples). Y-axis depicts antibody density or percentage labelled gametes (measured in SIFA); quartiles are indicated on the x-axis (Q1-Q4).

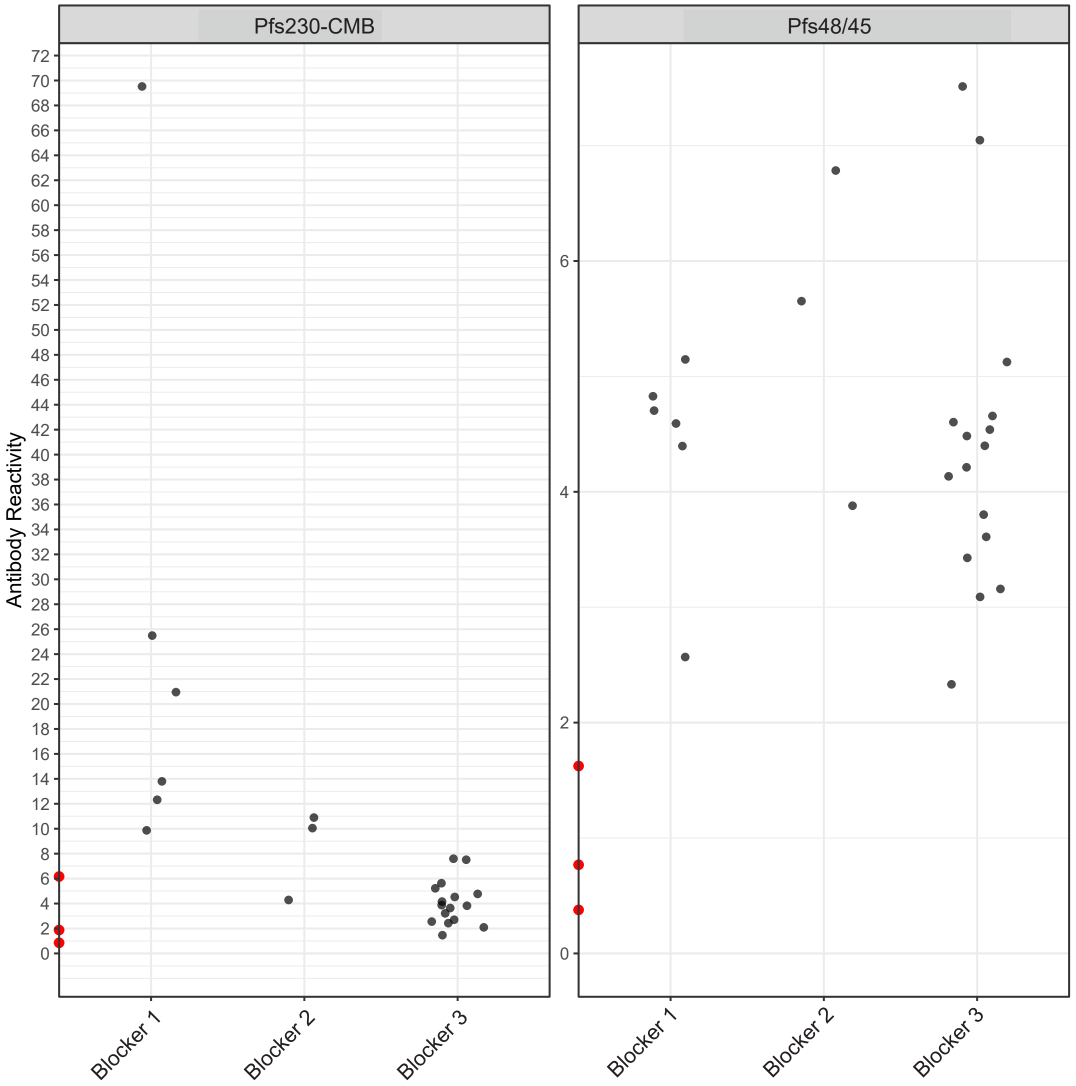

Supplementary Figure 5. Anti-Pfs230-CMB and anti-Pfs48/45 antibodies are detectable in functional transmission reducing activity (TRA) samples. Dot plots indicating antibodies against Pfs230-CMB (left panel) and against Pfs48/45 (right panel) using observations with functional TRA (≥80%) from three individuals that repeatedly showed functional TRA (blocker 1-3, n=25 observations, 3-16 per individual). The red dots on the y axis indicate the first quartile, median, and third quartile values per antibody response using samples from the 2013-2014 cross-section (n=313) for reference.

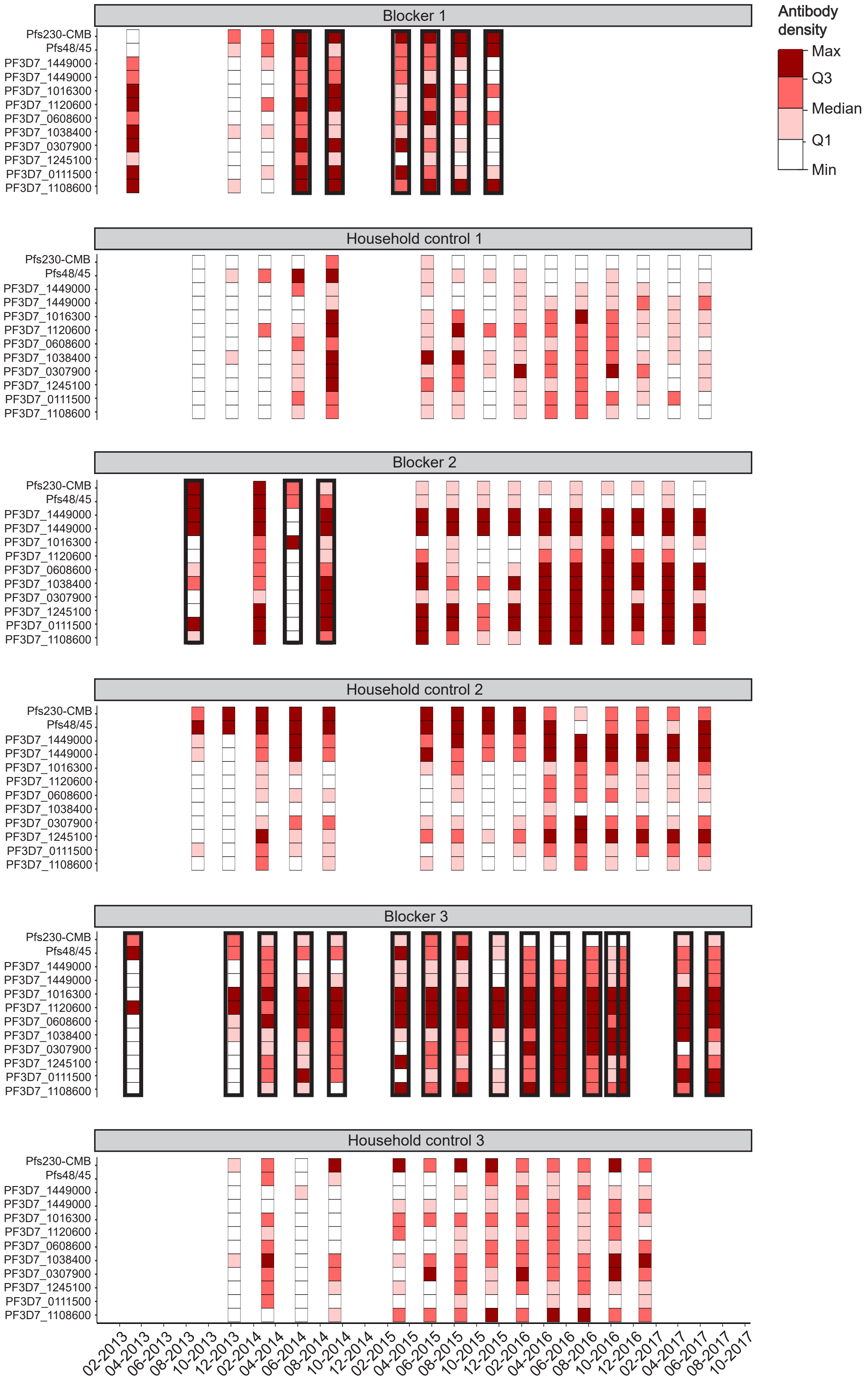

Supplementary Figure 6. Longitudinal patterns of transmission reducing activity (TRA) in relation to sexual-stage antibodies quantified by protein micro-array. Antibody levels are depicted in a heatmap over time for six individuals separately: three control individuals (n=43 samples) that never reached high-level TRA (≥80%) and three individuals that reached the high-level 80% TRA threshold more than once (i.e. blockers, n=39 samples). The maximum TRA of samples from control individuals was 71%. Using samples from all six individuals (n=82 samples), antibody densities were divided into quartiles using the 25th, 50th and 75th percentile value per antibody response. Samples with functional TRA are highlighted by heavier outlines. Sample collection dates are indicated on the x-axis.

Seroprevalence (%, n/N)	Age category < 5 years	5 – 11 years	≥ 18 years
**Asexual stage responses**			
Etramp5.Ag1	32.1 (186/579)	24.9 (328/1319)	41.5 (291/701)
GEXP18	8.9 (52/579)	15.1 (199/1319)	24.3 (170/701)
Rh22030	31.6 (183/579)	61.0 (804/1319)	60.6 (425/701)
AMA1	51.1 (296/579)	86.1 (1135/1319)	99.4 (697/701)
$MSP1_{19}$	43.7 (253/579)	48.7 (642/1319)	83.6 (586/701)
GLURP.R2	31.8 (184/579)	64.4 (850/1319)	98.6 (691/701)
**Sexual stage responses**			
Pfs230-CMB	37.5 (217/579)	58.3 (770/1321)	85.6 (599/700)
Pfs48/45	16.3 (94/578)	41.0 (541/1321)	73.8 (516/699)
Gametocyte lysate	75.5 (435/576)	93.9 (1231/1311)	99.1 (661/667)
PfHAP2-D2	6.6 (5/76)	27.0 (64/237)	47.8 (140/293)
PfMDV1	22.1 (17/77)	58.6 (139/237)	73.5 (216/294)
Pf77	7.8 (6/77)	40.9 (97/237)	69.7 (205/294)
Pfs47-Del2	36.8 (28/76)	47.3 (112/237)	69.4 (204/294)

Supplementary Table 1. Seroprevalence of antibodies against asexual and sexual stage responses. Asexual stage antibody responses were measured using a bead-based Luminex assay; sexual stage responses were measured using ELISA. Seroprevalence was calculated per age group as the number of seropositive routine visits divided by the total number of routine visits with seropositivity data.

* 100% = no change	Relative change in Ab density (%)<br>1-3 months since last infection			Relative change in Ab density (%)<br>3-12 months since last infection			Relative change in Ab density (%)<br>>12 months since last infection		
	**Median**	**IQR**	**N**	**Median**	**IQR**	**N**	**Median**	**IQR**	**N**
**Pfs230-CMB**	98.6	37.5-150.5	24	75.5	33.9-166.0	141	58.3	23.3-89.7	79
**Pfs48/45**	104.6	54.2-117.8	24	71.6	41.7-118.8	144	61.2	39.1-108.6	82
**Gametocyte lysate**	102.1	68.0-124.9	24	56.8	29.3-101.8	144	39.6	19.1-68.2	84
**Pfama1**	99.0	87.6-102.0	26	93.3	74.5-100.8	151	86.0	58.8-98.1	85
**Pfmsp119**	90.2	71.4-100.1	26	69.2	32.1-95.9	151	69.0	37.4-96.4	85
**Pfglurp.r2**	94.8	83.6-109.7	26	78.4	69.0-98.3	151	66.3	38.4-84.1	85
**Pfetramp5ag1**	91.1	62.7-116.6	26	66.1	35.5-97.3	151	51.9	23.1-94.0	85
**Pfgexp18**	91.3	79.6-109.6	26	69.6	45.3-92.9	151	65.2	46.2-99.1	85
**Pfrh22030**	89.1	72.5-109.3	26	66.4	41.8-94.0	151	59.2	29.9-84.3	85

Supplementary Table 2: Median antibody densities relative to the most recent parasite-positive time-point. Relative antibody densities were calculated per antibody response as the percent change in antibody density between each parasite-free time-point and its most recent parasite-positive time-point (i.e. antibody decay). The last parasite-positive time-point included visits with asymptomatic parasitemia (either sub-microscopic or microscopic) as well as clinical malaria episodes. The time since last parasite-positive time-point was categorized into groups of samples with recent (1-3 months since positivity), intermediate (3-12 months) or historic exposure (>12 months).

**Criteria ID**	**Genes matching criteria**	***Genes added***	**Gene cumulative total**	**Criteria for inclusion**	**Simplified criteria**
**1**	372	*372*	372	Gametocyte protein score >0 PLUS (presence of TM/SP/GO term indicating membrane OR surface expression (excluding mitochondrial proteins))	Protein moderately upregulated in gametocytes, and indication of membrane expression (excluding mitochondrial)
**2**	36	*35*	407	Zero protein evidence in any proteomic analysis, or evidence of translational repression in literature PLUS high gametocyte transcript score (>9.63) PLUS (presence of tm/sp/go term indicating membrane OR surface expression (excluding mitochondrial proteins))	Zero protein scores but gametocyte specific transcript (evidence of failure to detect protein, and/or mRNA storage/translational repression)
**3**	60	*29*	436	Gametocyte protein score >-10 (some evidence) PLUS (presence of GPI anchor [Gilson et al. Mol Cell Proteomics, 2006]) OR predicated export protein (Public domain).	Presence of protein in gametocytes, plus GPI anchor, or export association
**4**	30	*7*	443	Putatively exported by and specific to early or late gametocytes (Silvestrini et al. Mol Cell Proteomics, 2010).	Additional exported proteins specific to early gametocytogenesis
**5**	107	*75*	518	Gametocyte protein score of >9.69, regardless of protein structure/function, and an average peptide score of 9 in each gametocyte proteome database	Highly upregulated in gametocytes regardless of structure/function
**6**	43	*13*	531	Gametocyte specific proteins (Florens et al. Nature, 2002) seropositive in field sera in >50% of samples after transmission season OR with >20% seroprevalence increase after transmission season (Skinner et al. Infect Immun, 2015)	Gametocyte specific in the first gametocyte proteomic analysis (Florens et al. Nature, 2002) with evidence of immune recognition (Skinner et al. Infect Immun, 2015) (>50% samples after transmission season or >20% increase)
**7**	41	*7*	538	Gold standard gametocyte protein in list used to generate transcript and protein score	Gold standard gametocyte proteins
**8**	37	*23*	561	*A prioiri* selection. Markers of sexual stage exposure, TBV candidates, 6-cys proteins, proteins implicated in gamete fertility, and markers of asexual exposure	*A priori* selection: markers of asexual and sexual stage, vaccine candidates
**9**	45	*19*	580	Inclusion based on correlation with transmission blocking immunity (Stone et al.Nat Commun, 2018).	Inclusion based on association with transmission blocking immunity in our earlier work (Stone et al. 2018, Nat Commun).
**10**	47	*20*	**600**	Inclusion based on recognition by mice/immune sera and presence on giRBC surface (Dantzler et al. Sci Transl Med, 2019).	Inclusion based on presence and recognition on giRBC surface (early gametocytes) (Dantzler et al. Sci Transl Med, 2019).

Supplemental table 3. Selection criteria for genes expressed as proteins on the gametocyte protein microarray

			TRA category				
	Individuals (Total, N)	TRA observations (Total, N)	< -50% (N, %)	-50 – 9% (N, %)	10 – 49% (N, %)	50 – 79% (N, %)	≥ 80% (N, %)
**Cross-sectional blockers**	5	42	1 (2.4%)	4 (9.5%)	6 (14.3%)	5 (11.9%)	26 (61.9%)
**Cross-sectional controls**	54	314	12 (3.8%)	95 (30.3%)	146 (46.5%)	56 (17.8%)	5 (1.6%)

Supplementary Table 4. Transmission reducing activity (TRA) categories of samples from individuals with high level TRA (≥80%TRA, blockers) and without high level TRA (controls) in a 2013-2014 cross-sectional dataset. Additional samples from cross-sectional blockers (n=38; 0-15 per individual) and controls (n=260, 1-18 per individual) were selected for standard membrane feeding assays. Cross-sectional controls that showed high level TRA in any of the additional samples were considered as transmission blockers in later analyses.

Protein	Description	Gene ID	Location*	Marker	Function	Reference (PMID)
PfAMA1	Apical membrane antigen 1	PF3D7_1133400	Merozoite surface	Historical exposure	Erythrocyte invasion, immune evasion	17192270, 15542195, 21347343
PfMSP1_19	Merozoite surface protein 1-19	PF3D7_0930300	Merozoite surface	Historical exposure	Erythrocyte invasion, immune evasion	8078519, 12654798
GLURP R2	Glutamate-rich protein	PF3D7_1035300	Asexual, hepatic, gametocyte stages	Historical exposure	Erythrocyte invasion, merozoite development	7719909
MSP2 CH150/9	Merozoite surface protein 2 full-length [CH150/9]	PF3D7_0206800	Merozoite surface	Historical exposure	Erythrocyte invasion, immune evasion	16111789
MSP2 Dd2	Merozoite surface protein 2 full-length [Dd2]	PF3D7_0206800	Merozoite surface	Historical exposure	Erythrocyte invasion, immune evasion	16111789
Etramp 4 Ag 2	Early transcribed membrane protein 4 antigen (exon) 2	PF3D7_0423700	iRBC surface/PVM	Recent exposure	PVM integrity, immune evasion	12686607, 26216993
Etramp 5 Ag 1	Early transcribed membrane protein 5 antigen (exon) 1	PF3D7_0532100	iRBC surface/PVM	Recent exposure	PVM integrity, immune modulation	12686607, 32972398, 26216993
GEXP18	Plasmodium exported protein	PF3D7_0402400	Gametocyte surface	Recent exposure	Maturation of gametocytes	26216993
HSP40 Ag 1	Heat shock protein 40, antigen 1	PF3D7_0501100.1	iRBC/PVM	Recent exposure	Protein export, host cell remodeling	26216993
Hyp2	Plasmodium exported protein (hyp2)	PF3D7_1002000	iRBC/PVM	Recent exposure	Possible host cell remodeling, immune evasion	26216993
SBP1	Skeleton-binding protein 1	PF3D7_0501300	iRBC	Recent exposure	Erythrocyte invasion and remodeling	11087921, 21266965
CSP	Circumsporozoite protein	PF3D7_0304600	Sporozoite surface	RTS,S, liver stage exposure/protection	Hepatocyte invasion, immune evasion	23275094, 21604980, 33533814
PfSEA-1	Schizont egress antigen-1	PF3D7_1021800	iRBC	Protection	Schizont egress, iRBC membrane disruption	24855263, 28468980, 31288996
Rh2_2030	Reticulocyte binding protein 2 homologue a	PF3D7_1335400	Merozoite rhoptry	Protection	Erythrocyte invasion	11160005, 26833236
Rh4.2	Reticulocyte binding protein homologue 4 (RH4)	PF3D7_0424200	Merozoite rhoptry	Protection	Erythrocyte invasion	23028883, 26833236
Rh5.1	Reticulocyte binding protein homologue 5	PF3D7_0424100	Merozoite rhoptry	Protection	Erythrocyte invasion	18621009, 19000690, 21909261, 32359873, 22080952

EBA140 RIII-V	Erythrocyte binding antigen-140	PF3D7_1301600	Merozoite microneme	Protection	Erythrocyte invasion	20843207, 26833236, 24379273
EBA175 RIII-V	Erythrocyte binding antigen-175	PF3D7_0731500	Merozoite microneme	Protection	Erythrocyte invasion	20843207, 27644034, 16051144
EBA181 RIII-V	Erythrocyte binding antigen-181	PF3D7_0102500	Merozoite microneme	Protection	Erythrocyte invasion	20843207, 17087826
Pfs230 CMB	Plasmodium falciparum sexual stage antigen 230	PF3D7_0209000	Gametocyte surface	Potential transmission blocking	gametocyte development and fertility	38086855, 30824357, 8720173
Pfs 48/45 10 full length	Plasmodium falciparum sexual stage antigen 48/45 full length	PF3D7_1346700	Gametocyte surface	Potential transmission blocking	gametocyte development and fusion	36153317, 30237518, 39817720, 34642303
Pfs 48/45 10N	Plasmodium falciparum sexual stage antigen 48/45 N-terminal domain	PF3D7_1346700	Gametocyte surface	Potential transmission blocking	gametocyte development and fusion	36153317, 30237518, 39817720, 34642303
Pfs 48/45 10C	Plasmodium falciparum sexual stage antigen 48/45 central and C-terminal 6-Cys domain	PF3D7_1346700	Gametocyte surface	Potential transmission blocking	gametocyte development and fusion	36153317, 30237518, 39817720, 34642303
Pfs 48/45 6C	Plasmodium falciparum sexual stage antigen 48/45 C-terminal 6-Cys domain	PF3D7_1346700	Gametocyte surface	Potential transmission blocking	gametocyte development and fusion	36153317, 30237518, 39817720, 34642303
gSG6	Anopheles gambiae salivary gland protein 6	13537666	An. gambiae salivary gland	An.gambiae saliva exposure	Mosquito salivary protein	12062411, 21794142, 21437289, 22768250, 38906949
Tetanus toxoid	Tetanus toxoid vaccine	N/A	N/A	Internal positive vaccine control	N/A	
GST	Glutathione S-Transferase tag	N/A	N/A	Expression tag cross-reactivity control	N/A	

Supplementary Table 5. Panel of antigens included in Luminex assays. *iRBC, infected red blood cell; PVM, parasitophorous vacuole membrane
